# Trace Conditioning in *Drosophila* Induces Associative Plasticity in Mushroom Body Kenyon Cells and Dopaminergic Neurons

**DOI:** 10.3389/fncir.2017.00042

**Published:** 2017-06-20

**Authors:** Kristina V. Dylla, Georg Raiser, C. Giovanni Galizia, Paul Szyszka

**Affiliations:** Department of Biology, Neurobiology, University of KonstanzKonstanz, Germany

**Keywords:** *Drosophila*, dopaminergic neurons, Kenyon cells, mushroom body, trace conditioning, associative plasticity, memory acquisition, calcium imaging

## Abstract

Dopaminergic neurons (DANs) signal punishment and reward during associative learning. In mammals, DANs show associative plasticity that correlates with the discrepancy between predicted and actual reinforcement (prediction error) during classical conditioning. Also in insects, such as *Drosophila*, DANs show associative plasticity that is, however, less understood. Here, we study associative plasticity in DANs and their synaptic partners, the Kenyon cells (KCs) in the mushroom bodies (MBs), while training *Drosophila* to associate an odorant with a temporally separated electric shock (trace conditioning). In most MB compartments DANs strengthened their responses to the conditioned odorant relative to untrained animals. This response plasticity preserved the initial degree of similarity between the odorant- and the shock-induced spatial response patterns, which decreased in untrained animals. Contrary to DANs, KCs (α'/β'-type) decreased their responses to the conditioned odorant relative to untrained animals. We found no evidence for prediction error coding by DANs during conditioning. Rather, our data supports the hypothesis that DAN plasticity encodes conditioning-induced changes in the odorant's predictive power.

## Introduction

Associative learning enables animals to anticipate negative or positive events. The neural mechanisms of associative learning are commonly studied in classical conditioning paradigms, in which animals are trained to associate a cue (conditioned stimulus; CS) with a punishment or reward (unconditioned stimulus; US; Pavlov, 1927). In the standard conditioning paradigm CS and US overlap in time, while in the trace conditioning paradigm there is a temporal gap between the CS and US. During both standard conditioning and trace conditioning, the US is mediated by dopaminergic neurons (DANs), in animals as diverse as monkeys and fruit flies (Shuai et al., 2011; Dylla et al., 2013; Schultz, 2013; Waddell, 2013).

Genetic tools for monitoring and manipulating neuronal activity in the fruit fly *Drosophila melanogaster* promoted the understanding of the neural mechanisms of dopamine-mediated learning. Those mechanisms are well-described for standard “odor—shock conditioning” in *Drosophila*, in which an olfactory CS is paired with a temporally overlapping electric shock US (Quinn et al., 1974; Tully, 1984; Pitman et al., 2017). During conditioning, an odor—shock association is formed in the mushroom body (MB) neuropil. The intrinsic neurons of the MB, the Kenyon cells (KCs), receive olfactory input in the MB-calyx and project to the vertical (α and α'), and the medial (β, β', and γ) MB-lobes. During odor—shock conditioning, the olfactory CS activates an odorant-specific KC population (Murthy et al., 2008; Turner et al., 2008), and the electric shock US activates DANs that innervate the MB-lobes (Riemensperger et al., 2005; Mao and Davis, 2009; Aso et al., 2010, 2012). In KCs, the CS-induced increase in intracellular calcium and the US-(dopamine)-induced second messengers synergistically activate an adenylyl cyclase (Duerr and Quinn, 1982; Dudaí et al., 1983; Tomchik and Davis, 2009; Gervasi et al., 2010), which alters the synaptic strength between KCs and MB output neurons (MBONs). This change in KC-to-MBON synapses is thought to encode the associative odor memory (Dubnau et al., 2001; McGuire et al., 2001; Schwaerzel et al., 2003; Séjourné et al., 2011; Pai et al., 2013; Zhang and Roman, 2013; Aso et al., 2014b; Bouzaiane et al., 2015; Cohn et al., 2015; Hige et al., 2015a; Owald et al., 2015).

The MB-lobes are divided into 15 compartments (α1–3, β1–2, α'1–3, β'1–2, and γ1–5), each of which is innervated by a distinct population of DANs and MBONs (Tanaka et al., 2008; Aso et al., 2014a). These compartments constitute functional units, which are involved in different forms of associative learning (Tanaka et al., 2008; Séjourné et al., 2011; Pai et al., 2013; Plaçais et al., 2013; Aso et al., 2014a,b; Bouzaiane et al., 2015; Cohn et al., 2015; Masek et al., 2015; Hige et al., 2015a,b; Owald et al., 2015). In compartments such as γ1, γ2, and β2, DANs mediate electric shock reinforcement (Aso et al., 2010, 2012; Qin et al., 2012). Besides mediating reinforcement during classical conditioning, *Drosophila* DANs are involved in long-term memory formation (Plaçais et al., 2012), forgetting (Berry et al., 2012, 2015), extinction learning and memory reconsolidation (Felsenberg et al., 2017), and in integrating internal states with memory and sensory processing (Krashes et al., 2009; Shuai et al., 2011; Liu et al., 2012; Ueno et al., 2012; Alekseyenko et al., 2013; Lin S. et al., 2014; Cohn et al., 2015; Lewis et al., 2015; Musso et al., 2015; Sitaraman et al., 2015; Nall et al., 2016). A single DAN can even serve different functions, for example, PPL1-γ1pedc (also referred to as MB-MP1) signals reinforcement (Aso et al., 2010; Aso and Rubin, 2016), gates long-term memory formation (Plaçais et al., 2012; Musso et al., 2015), and controls state-dependent memory retrieval (Krashes et al., 2009).

The functional complexity of *Drosophila* DANs is further increased by the fact that DANs show learning-induced associative plasticity: they increase their response to the CS during classical conditioning (Riemensperger et al., 2005). Mammalian DANs also increase their CS-induced responses during classical conditioning (Schultz et al., 1993, 1997). In addition, they decrease their response to the US, and when a predicted US does not occur, they decrease their activity below baseline level (Schultz et al., 1993, 1997). This pattern of response plasticity in mammalian DANs is compatible with the hypothesis that animals only learn to associate a CS with a US, when the US occurs unpredictably (Kamin, 1969; Rescorla and Wagner, 1972). Thus, mammalian DANs appear to encode this prediction error (Schultz et al., 1997). In *Drosophila*, however, DANs do not change their response to the US (Riemensperger et al., 2005). Therefore, *Drosophila* DANs appear to encode the US prediction by the CS rather than encoding the US prediction error during classical conditioning (Riemensperger et al., 2005). It is not clear, whether classical conditioning in insects is driven by US prediction error. There is evidence for prediction error-driven conditioning in crickets (Terao et al., 2015), but there is also a controversy about whether or not blocking—a failure to learn, when the US is already predicted by another CS (Kamin, 1969)—occurs in honey bees (Smith and Cobey, 1994; Gerber and Ullrich, 1999; Hosler and Smith, 2000; Guerrieri et al., 2005).

**Figure 1:**
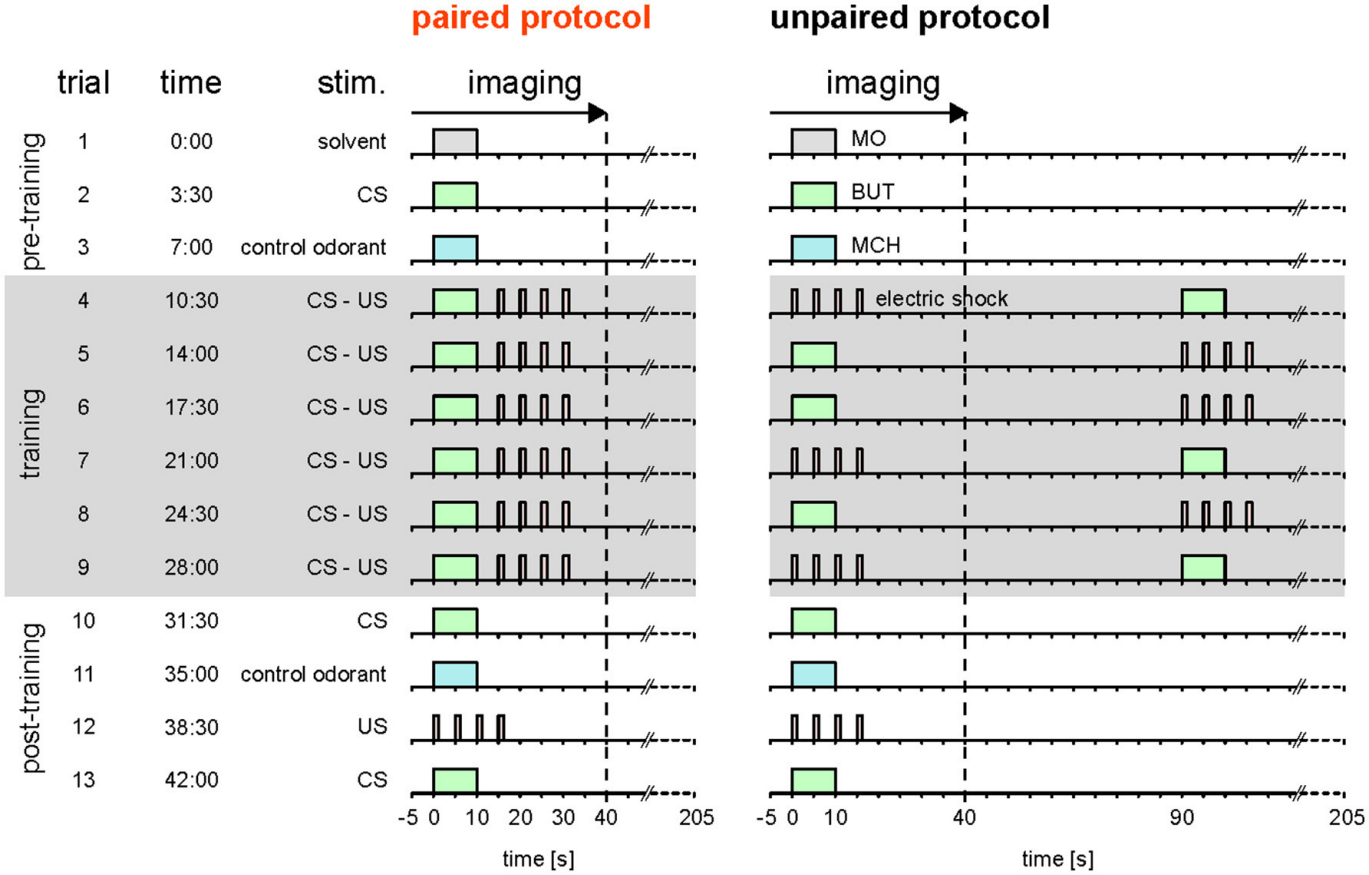
Stimulation protocols. Paired and unpaired stimulation protocol. Both protocols were identical except for the training phase. Pre-training (trial 1–3): 10-s-long pulses of the solvent (MO; gray), the olfactory CS (BUT; green), and the control odorant (MCH; blue) were applied. Training (trial 4–9, shaded in gray): each of the six training trials consisted of a 10-s-long CS pulse and four 1.5-s-long 90 V US pulses (electric shock; red). The interval between the onsets of CS and US was 15 s in the paired protocol and 90 s in the unpaired protocol. In the unpaired group, the sequence of CS and US was pseudorandomized. Note that in both groups there was a stimulus-free gap between CS and US. Post-training (trial 10–13): CS, control odorant and US were followed by a last CS presentation at the end of the protocol to detect a possible run-down of calcium signals. The inter-trial interval was 210 s. Calcium imaging was performed during the first 45 s of each trial. Therefore, for the unpaired group only the first stimulus in each trial was recorded. The time of trial onsets is given in minutes. Each protocol lasted 45.5 min.

Here, we reassessed the hypothesis that *Drosophila* DANs do not encode the prediction error during classical conditioning (Riemensperger et al., 2005). Different to Riemensperger et al. (2005) who pooled DAN activity across the mushroom body lobes, we differentiated between DAN types that innervate different compartments of the MB lobes. Moreover, instead of using standard conditioning, we used trace conditioning with a 5 s gap between the CS and the US (Figure 1; Galili et al., 2011), which allowed us to more precisely distinguish between responses to either the CS or the US.

We monitored CS- and US-induced calcium responses before, during, and after odor—shock trace conditioning in DANs and in their synaptic partners, the KCs. To separate associative from non-associative effects caused by the conditioning procedure, we compared the effect of paired CS-US presentations against isolated (unpaired) CS and US presentations. We found that during trace conditioning, DANs increased and KCs decreased their CS-induced responses relative to the unpaired control group. The occurrence and strength of this response plasticity varied across MB compartments. US-induced DAN responses, however, did not change, and neither did DAN activity change during omission of a predicted US. These data support the hypothesis that DANs encode predictive power of the CS, but not the US prediction error (Riemensperger et al., 2005). We discuss the implications of these data for the neural substrate of sensory odor memories (traces) and the MB circuitry.

## Materials and methods

See Supplemental Experimental Procedures for more details.

### Flies and fly preparation

**Figure 2:**
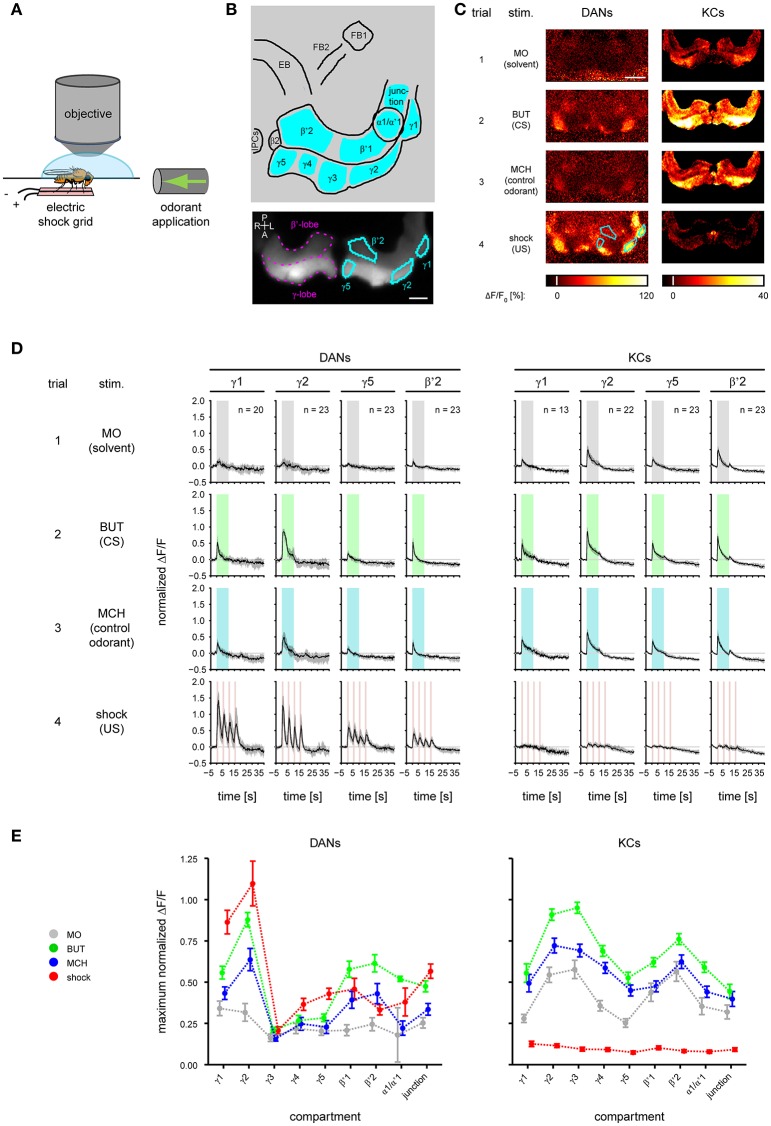
Odorant- and electric shock-induced responses in dopaminergic neurons (DANs) and Kenyon cells (KCs) differ between mushroom body (MB) compartments. **(A)** During calcium imaging electric foot shock and odorants were applied to the fly. **(B)** Top: schematic view of the analyzed regions. FB, fan-shaped body; EB, ellipsoid body; IPCs, insulin-producing cells. Nine MB compartments (indicated by cyan) were analyzed in both KCs and DANs. Bottom: DsRed raw fluorescence image with the MB β'- and γ-lobe (magenta) indicated in the right brain hemisphere. Four exemplary MB compartments γ1, γ2, γ5, and β'2 (cyan) are indicated in the left hemisphere. Dorsal view; P, posterior; L, left; A, anterior; R, right. Scale bar: 40 μm. **(C)** Color-coded activity patterns obtained for stimulations with odorants and electric shock in DANs (*TH*>*GCaMP3* fly) and KCs (*OK107*>*GCaMP3* fly) in the unpaired group prior to training (trial 1–4). The four exemplary MB compartments are identical to those in **(B)**. Scale bar: 80 μm. **(D)** Response traces obtained for stimulation with odorants and electric shock in DANs and KCs in γ1, γ2, γ5, and β'2. Traces are normalized to the strongest response amplitude induced by the first BUT (CS) presentation in any region of interest, and show the median and quartiles over all flies in the unpaired group [number of flies (n) is indicated in the figure]. **(E)** Maximum response obtained for stimulation with odorants and electric shock in DANs and KCs in nine compartments. All curves represent the mean and SEM, *n* = 2–23.

For imaging DANs, we crossed females homozygous for both *UAS-GCaMP3* (Tian et al., 2009) and *TH-GAL4* (Friggi-Grelin et al., 2003) with males homozygous for *mb247-DsRed*; *mb247-DsRed* (Riemensperger et al., 2005) so that DsRed expression in the MBs could be used as a morphological landmark. We refer to the F1 flies as *TH*>*GCaMP3*. To drive GCaMP3 expression in the KCs we crossed homozygous male *UAS-GCaMP3* flies with homozygous female *OK107-GAL4* flies (Connolly et al., 1996). We refer to the F1 flies as *OK107*>*GCaMP3*. For imaging, we anesthetized a single fly on ice, fixed it in a holder, opened the fly head dorsally and covered the preparation with saline (Figure 2A).

### Stimuli and stimulus control

**Figure 3:**
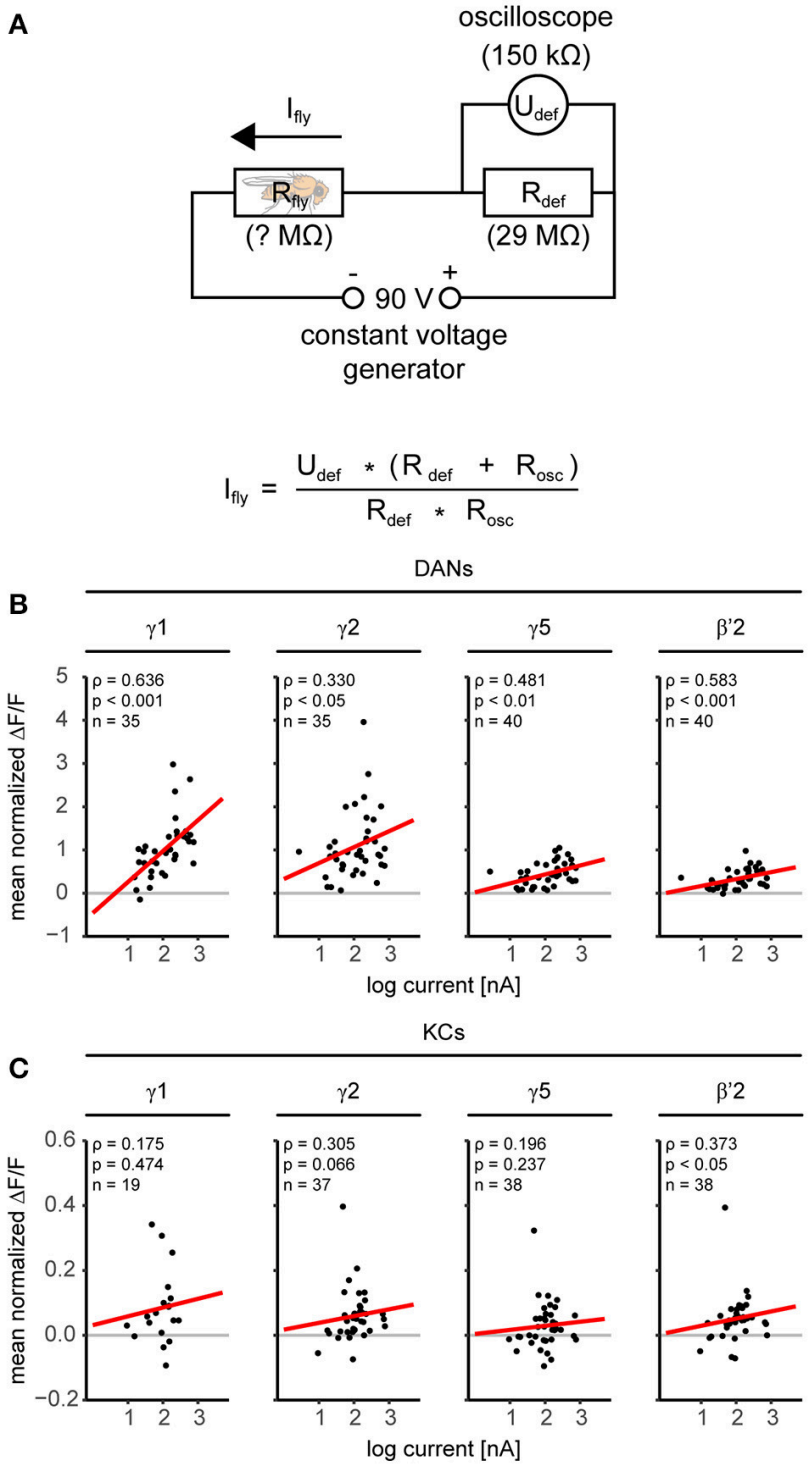
Dopaminergic neurons are more sensitive to the electric shock strength than KCs. **(A)** Electric circuit for monitoring the current flow through the fly during electric shock application. The voltage generator provided constant 90 V pulses. The current flow through the fly (I_fly_) was determined by measuring with an oscilloscope (*R* = 150 kΩ) the voltage (U_def_) over a defined resistor (*R*_def_ = 29 MΩ). **(B)** Relationship between electric shock-induced responses in DANs and individual current flow. Responses correlated with current in all four compartments in DANs (red regression line). Results of a Spearman rank correlation test and the number of flies (n) are indicated in the figure. **(C)** Same analysis as in B, but for KCs. Responses in KCs were small as compared to DANs, nevertheless responses correlated with current in β'2. For all analyzed regions see Supplementary Table 2.

We applied electric shocks (four 1.5 s long 90 V pulses) to the fly's legs by placing the fly on a custom-build copper grid (Figure 2A and Supplementary Figure 4B). We recorded the shock strength received by an individual fly using a bridge circuit (sampling rate: 16 kHz; Figure 3A, Supplementary Figure 4C, and Supplementary Table 2). We used 1-butanol (BUT) and 4-methylcyclohexanol (MCH) diluted in mineral oil (MO; BUT 1:500, MCH 1:1,000) as odorant stimuli, which we presented as 10 s long stimuli with a custom-build stimulator (Szyszka et al., 2011). We measured the dynamics of the odorant stimuli with a photo ionization detector (miniPID, Aurora Scientific Inc.). Rapid odorant stimulus termination (Supplementary Figure 4A) allowed us to distinguish between responses to the olfactory CS and to the electric shock US.

### Calcium imaging

We measured the fluorescence of GCaMP3 and DsRed at a sampling rate of 5 Hz using a confocal laser scanning microscope, equipped with a 20 × water-immersion objective. Note that in some physiological paradigms animals are pre-exposed to the stimuli until the induced neuronal response strength becomes stable (Hige et al., 2015a). Since stimulus pre-exposure is not common in the behavioral odor—shock conditioning paradigm (Tully and Quinn, 1985), we did not pre-expose our flies to the applied stimuli.

### Data analysis

#### Imaging data

First, we corrected the movement in the confocal imaging data within each trial by registering the mb247-DsRed of each frame to a common reference, and applying the obtained transform to the GCaMP3 signal. Then, we identified the MB compartments visually according to the studies by Tanaka et al. (2008) and Aso et al. (2014a) as regions of interest, based on the GCaMP3- and DsRed-expression for KCs and DANs, respectively. Note, that we also refer to merged compartments like “α1/α'1” and regions like the “junction” as compartments in this study. From each frame, we subtracted the background fluorescence before odorant onset (F_0_, mean of frames 3–24) to get ΔF/F_0_. Because the signal amplitude varied between flies, we normalized the ΔF/F_0_-traces within each fly to the maximum of the BUT-induced response in trial 2 in the strongest responding compartment. The normalized ΔF/F_0_-values are referred to as “response trace.”

#### Color-coded images (Figures 2C, **5A**)

For color-coded images of spatial activity patterns, we calculated the average percentage change in the response traces during stimulus application.

#### Changes in response trace (Figure 4A)

To visualize changes in the stimulus-induced responses during training, we subtracted response traces before training from response traces during or after training. Above the response traces we plotted color-coded *p*-values (Wilcoxon test) obtained for each single frame to quantify differences between paired and unpaired group.

**Figure 4:**
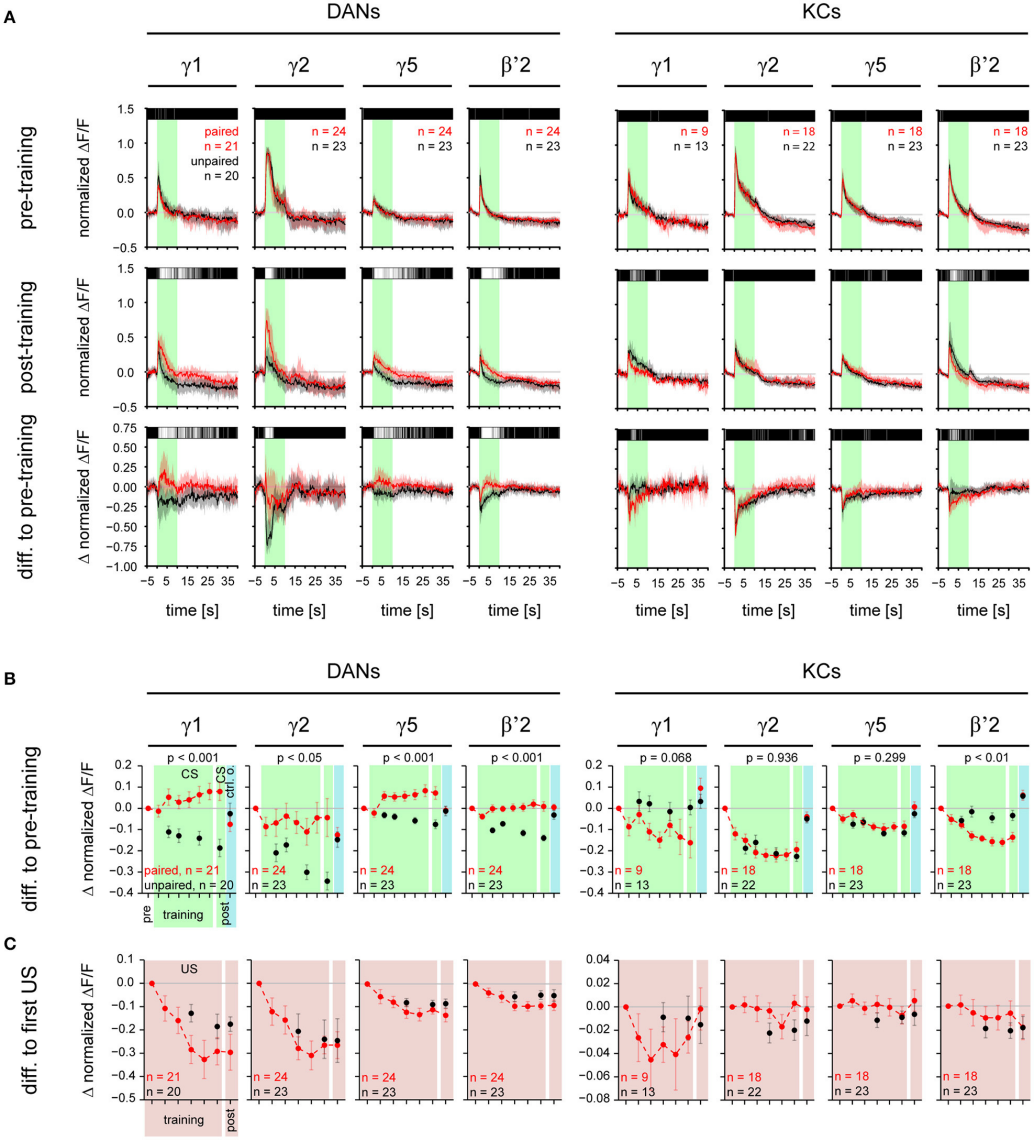
CS-induced responses change during training in a compartment-specific manner in DANs and KCs. **(A)** Normalized DAN and KC response traces obtained for stimulations with the CS for γ1, γ2, γ5, and β'2 in the paired (red) and the unpaired (black) group, before and after training. Top: during pre-training the responses to the CS did not differ between the paired and the unpaired group. Middle: Post-training, in DANs in all four compartments the response to the CS was stronger in the paired than in the unpaired group. In KCs, in β'2 the response to the CS was weaker in the paired than in the unpaired group. Bottom: difference in response traces between post- and pre-training. Positive values reflect an increase, negative values a decrease in response strength after training. Traces represent the median and quartiles [number of flies (n) is indicated in the figure]. The bar code above the traces indicates the *p*-value obtained for each frame from a Wilcoxon test between paired and unpaired group (black: *p* ≥ 0.05, dark gray: *p* < 0.05, light gray: *p* < 0.01, white: *p* < 0.001). All traces of the paired group are shown in Supplementary Figures 1, 2. **(B)** CS-induced response strength in DANs and KCs during the six training trials and the post-training. The pre-training response strength to the CS or the control odorant was subtracted from each value. DANs: During training the CS-induced response strength increased in the paired group relative to the unpaired group in all four compartments (*p*-values are indicated in the figure; mixed-effect model for repeated-measures ANOVA). Post-training the response strength induced by the CS was higher in the paired than in the unpaired group in all four compartments. The response strength induced by the control odorant (blue background) did not differ between the paired and unpaired group (Supplementary Figure 3A). KCs: During training the CS-induced response strength decreased in the paired group relative to the unpaired group in β'2, but not in the other three compartments. Post-training the CS-induced response strength was lower in the paired than in the unpaired group in β'2 only. The response strength induced by the control odorant (blue background) did not differ between the paired and unpaired group (Supplementary Figure 3B). **(C)** US-induced response strength in DANs and KCs during the six training trials and the post-training. The first US-induced response strength was subtracted from each value. In both, DANs and KCs, US-induced responses did not differ between the paired and the unpaired groups. All values represent the mean and SEM. For all analyzed regions see Supplementary Figures 3A,B. Note, that both the paired and unpaired protocol comprise six CS (and six US) presentations. However, in the unpaired group, we recorded DAN and KC activity only during three CS and three US, in order to keep the total imaging exposure times (and thus bleaching) for the paired and the unpaired groups equal. For statistics we used only those trials which have been recorded in both the paired and unpaired group.

#### Changes in response strength (Figure 4B, Supplementary Figure 3)

We quantified training-induced changes in response strength by averaging the response trace over the time of stimulus application (10 s for odorants and 4 × 1.5 s for electric shock). To correct for differences in the baseline fluorescence, we calculated the change in response strength relative to the frame prior to the onset of odorant or electric shock pulse. We calculated the difference between the respective training or test trial and the corresponding pre-training trial (trial 2 for the CS, trial 3 for the control odorant, and trial 4 for the US). The calculated value is referred to as “response strength.”

First, we analyzed left and right brain hemispheres separately and tested for a significant difference between hemispheres using a linear mixed-effect model on the data (R: “lme” function). In some regions, the US-induced DAN responses in the unpaired protocol and the CS-induced KC responses in the paired protocol differed between hemispheres (Supplementary Table 1). We selected the hemisphere in which the underrepresented MB-compartment γ1 was visible for further analysis.

#### Associative plasticity (**Figure 6A**)

We quantified associative plasticity as the difference in response strength between paired and unpaired group.

#### Spatial activity patterns (Figure 5B and Supplementary Figure 6)

We compared the spatial activity pattern induced by a stimulus in the brain of an individual fly over trials. To this end, we calculated the normalized mean response strength during stimulus application in each of the nine MB compartments shared between DANs and KCs. We used the mean response strength values as components of a 9-dimensional vector in a 9-dimensional space. The dissimilarity between two spatial activity patterns was determined by the Euclidean distance and by the angle (φ) between the two respective vectors ($\vec{\alpha }$ and $\vec{\beta }$).

$$\begin{array}{l} \text{cos}\varphi =\frac{\vec{\alpha }\;\circ \;\vec{\beta }}{\left||\vec{\alpha }||\;||\vec{\beta }|\right|} \end{array}$$

**Figure 5:**
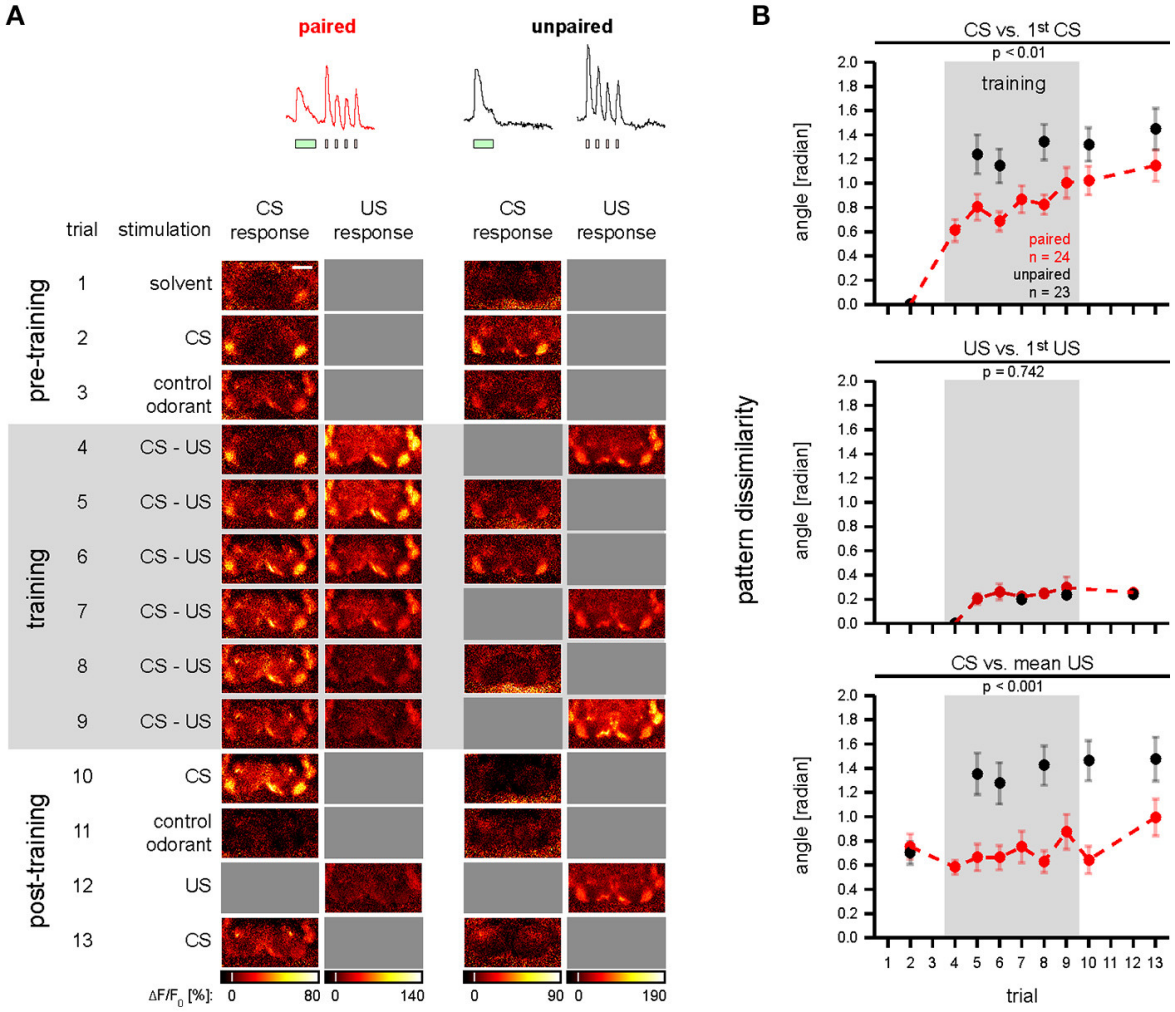
Odor—shock conditioning affects CS-induced spatial activity patterns in DANs. **(A)** Single animal examples showing color-coded images of responses induced during conditioning in single *TH*>*GCaMP3* flies of the paired and unpaired group. Gray squares indicate non-availability of data due to the experimental protocol. Scale bar: 80 μm. **(B)** Dissimilarity of spatial activity patterns in DANs was quantified as the angle between vectors that comprise the response strengths of all nine compartments. CS vs. 1st CS: during training the CS-induced spatial activity patterns became dissimilar to the pre-training spatial activity pattern. This effect was stronger in the unpaired (red) than in the paired group (black). US vs. 1st US: during training the US-induced spatial activity patterns became dissimilar to the first US-induced spatial activity pattern in the paired and unpaired group, however, there was no difference between the paired and the unpaired group. CS vs. mean US: during training the CS-induced spatial activity patterns became dissimilar to the mean US-induced activity pattern in the unpaired group, but not in the paired group. All traces represent the mean and SEM [*p*-values and number of flies (n) are indicated in the figure; mixed-effect model for repeated-measures ANOVA]. See Supplementary Figure 6 for KC data and for a pattern analysis with Euclidean distances.

#### Software

For controlling the electric shock application we used software written by Stefanie Neupert, University of Konstanz, for cRIO-9074, module NI-9403, in LabVIEW 2011 SP1 (National Instruments). To remove movement artifacts from the imaging data, across-channel image registration was performed using a custom elastix-based python toolkit (source code available at https://github.com/grg2rsr/xyt_movement_correction) and custom-written routines in IDL (Research Systems Inc.). Further, data processing and analysis we conducted in R (version i386 3.1.2, R Core Team, 2014) using custom-written routines.

#### Statistics

To meet the criteria for parametric statistical methods we used a Box-Cox transformation on the DAN data to achieve normal distribution. We tested for differences over training trials, between hemispheres, between experimental groups, and between paired and unpaired group using linear mixed-effect models. We performed repeated-measures ANOVAs on the linear mixed-effect models fitted to the data. We provide detailed information on the models and ANOVAs in Supplementary Table 1. For statistics, we excluded trials in which the corresponding stimulus presentation was not recorded in the unpaired group. Throughout the paper we indicate ^*^p < 0.05, ^**^p < 0.01, and ^***^p < 0.001, not significant *p* ≥ 0.05.

## Results

To record the CS- and US-induced responses in both DANs and KCs during odor—shock trace conditioning (Figure 1), we performed calcium imaging during which we applied odorants and electric foot shocks (Figure 2A and Supplementary Figure 4B). We recorded from both DANs and KCs in nine compartments of the MB-lobes (α1/α'1, β2, β'1–2, and γ1–γ5, cyan in Figure 2B). For imaging DANs, we used the morphologically and physiologically well-characterized driver line *TH-GAL4* (Friggi-Grelin et al., 2003; Mao and Davis, 2009) to drive expression of the fluorescent calcium sensor GCaMP3. *TH-GAL4* drives expression in DANs such as the protocerebral posterior lateral 1 (PPL1) cluster DANs and the protocerebral anterior medial (PAM) cluster DANs. Each of these DANs innervates one to two compartments in the medial MB-lobes: One to two PPL1-γ1pedc DANs and one PPL1-γ1 DAN in γ1, one PPL1-γ2α′1 DAN in γ2 and α'1, and 12 PAM DANs of which three innervate β2β'2a, while the remaining DANs innervate either β'2 or γ5 (Figures 2B,C; Mao and Davis, 2009; Aso et al., 2010, 2014a). Furthermore, we recorded from *TH-GAL4*-labeled PAM DANs in γ3, γ4, and β'1 (Figure 2E and Supplementary Figure 1; Pech et al., 2013). Note, that *TH-GAL4* covers only a small subpopulation of the about 120 PAM neurons that innervate the medial lobe (Aso et al., 2012; Pech et al., 2013).

To image KCs, we used *OK107*>*GCaMP3* flies. Unlike DANs, individual KCs send their axons across all compartments of a specific lobe [for instance: in the γ-lobe from γ1 to γ5 (Cohn et al., 2015), in the β'-lobe from β'1 to β'2; Figures 2B,C]. Thus, in our study the KC response of a compartment reflects the summed response of several hundreds of KCs.

Throughout the paper we show exemplary DAN and KC responses for the MB compartments γ1, γ2, γ5, and β'2 (Figure 2B). The complete data is shown in the supplement.

### DAN and KC responses to odorants and electric shock differ across MB compartments

First, we measured the DAN and KC responses to odorants and electric shock prior to training (Figure 1, trial 1–4, unpaired group). In DANs and KCs, odorants and electric shocks induced calcium responses across the MB compartments (Figure 2C). Both response dynamics and amplitudes differed between DANs and KCs, and across MB compartments (Figure 2D, Supplementary Figures 1, 2). Odorants induced DAN responses and KC responses in all MB compartments (Figure 2E). In general, responses were strongest to BUT (1-butanol), weaker to MCH (4-methylcyclohexanol) and weakest to MO (the solvent mineral oil). KCs in the β- and β'-lobe showed off-responses to the offset of odorants (Figure 2D and Supplementary Figure 2). KCs responded stronger to MO than DANs (Figure 2E). DANs responded stronger to electric shocks than KCs (Figures 2C–E), although both fly lines encountered equal shock strength (Supplementary Figure 4C). Note however, that the responses were normalized to the BUT responses, and that individual KCs could respond stronger to shock than to odorants. DAN responses to electric shock increased logarithmically with the electric current flow through the fly [linear regression line slopes ranged from 0.024 to 0.715 ΔF/F per log (nA) in α1/α'1 and γ1, respectively; Figure 3B and Supplementary Table 2], except in the MB-compartment α1/α'1, and were strongest in γ1 and γ2 (Figure 2E). There was a positive correlation between KC response strength and received current in the β'-lobe and in the γ3 compartment (Figure 3C and Supplementary Table 2).

### Associative plasticity of CS-induced—but not US-induced—DAN and KC responses

To investigate the effect of odor—shock trace conditioning on DAN and KC responses to the CS and to the US, we combined odor—shock trace conditioning with calcium imaging (Figure 1). To this end, we adopted the trace conditioning protocol from Galili et al. (2011). As Galili and colleagues, we used BUT as the only CS, because trace conditioning with BUT yielded the best results in behavioral experiments. Flies of the paired group received six CS-US pairings, whereas flies of the unpaired control group received six unpaired CS and US presentations. After the training, we recorded responses to the CS, MCH, and the US alone in both groups. The unpaired group served as a control for non-biological effects of the experimental procedure (bleaching of GCaMP, changes in stimulus strength; Supplementary Figures 4, 5), as well as for non-associative effects of conditioning (sensitization, habituation, pseudo-conditioning; Tully, 1984). However, note that our experimental design does not allow us to differentiate between the proportional contribution of non-biological and non-associative effects to the measured neuronal response strength. As flies of the paired and the unpaired group received the same number of CS and US, but with a different stimulus timing, differences in DAN and KC responses between the paired and the unpaired group reflect associative plasticity.

**Figure 6:**
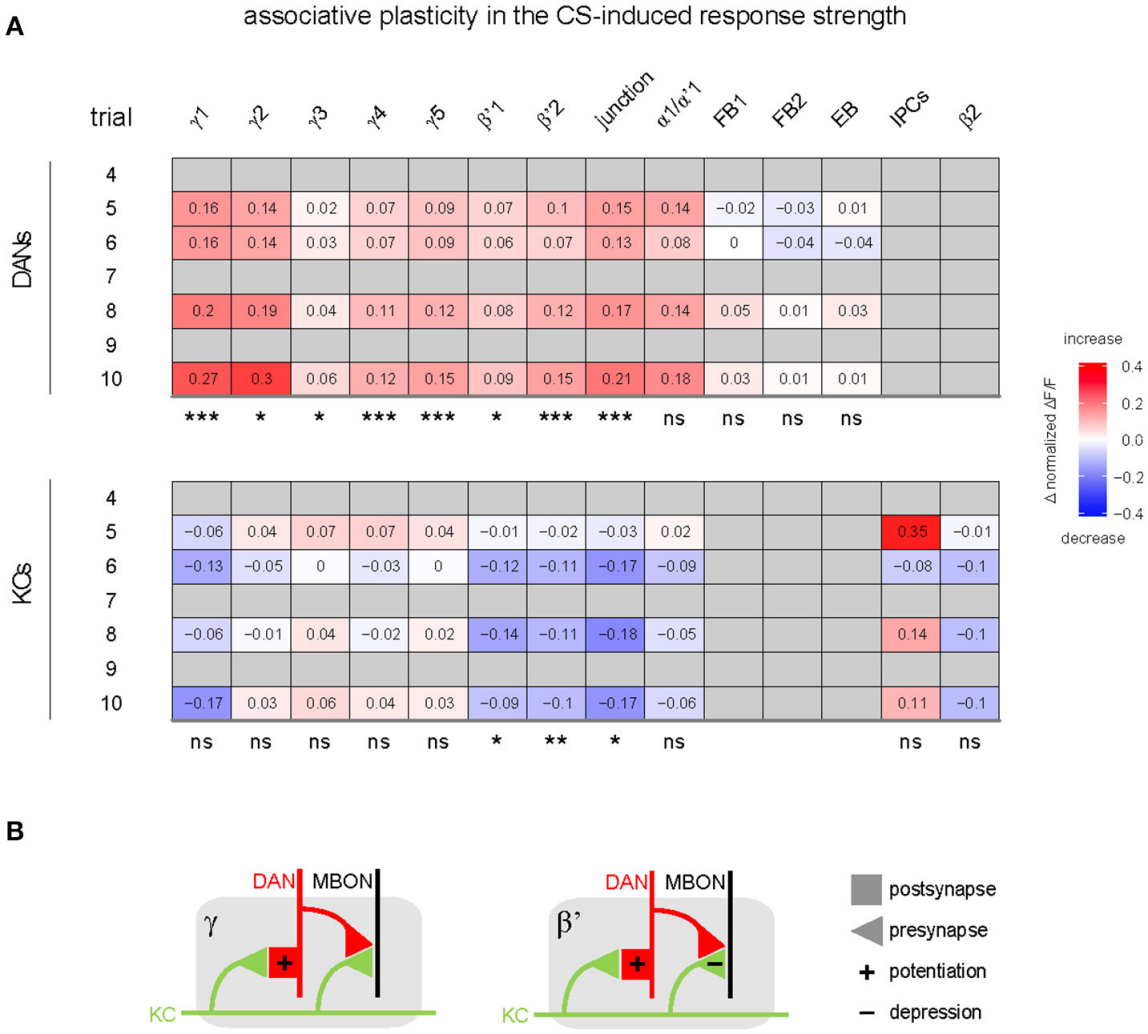
Summary of associative effects of odor—shock conditioning in DANs and KCs. **(A)** Values and color code show the difference in response strength to the CS between the paired and unpaired group for the different MB compartments and other regions innervated by either DANs (top), or KCs and IPCs (bottom; differences were calculated on the post-training response strength plotted in Figure 4B and Supplementary Figures 3A,B). Associative effects were defined as significant difference between the paired and the unpaired group (statistical significances are indicated below each table). In DANs, odor—shock conditioning induced an associative increase in the response to the CS in most compartments. In KCs, odor—shock conditioning induced an associative decrease in the response to the CS in three compartments (*n* = 2–24; mixed-effect model for repeated-measures ANOVA). Non-availability of data is indicated by gray. **(B)** Hypothetic circuit model of associative plasticity induced by odor—shock trace conditioning in β'- and γ-compartments. KC axons (green) traverse a compartment (gray) of either the β'- or γ-lobe. Each compartment is innervated by compartment-specific DANs (red) and mushroom body output neurons (MBONs; black). During trace conditioning, odor—induced KC and shock-induced DAN activity induce postsynaptic potentiation at the KC-to-DAN synapse, and induce synaptic depression in β'-KCs, but not in γ-KCs.

DAN responses to the CS increased in the paired relative to the unpaired group (Figures 4A,B and Supplementary Figure 3A) in eight out of nine MB compartments (γ1–5, β'1–2, and junction), but neither in the ellipsoid body nor in the fan-shaped body (Figures 4, 6A, and Supplementary Figure 3A). This training-induced associative plasticity in DANs became visible after the first training trial (trial 5 in Figure 1). We did not detect a correlation between changes in the CS-induced response strength and the US strength that a fly received in each trial (Supplementary Figure 3E).

In addition to the relative increase of CS-induced DAN responses in the paired compared to the unpaired group, there was an increase in the absolute DAN response strength from the first training trial to the post training trial in three of the γ-lobe compartments [mixed-effect model for repeated-measures ANOVA, γ1: *F*_(1, 20)_ = 4.39, *p* < 0.05; γ4: *F*_(1, 22)_ = 6.23, *p* < 0.05; γ5: *F*_(1, 23)_ = 6.67, *p* < 0.05].

In contrast to DANs, KC responses to the CS decreased in the paired relative to the unpaired group, and this decrease occurred in three out of nine MB compartments (β'1–2, and junction; Figures 4A,B, 6A, and Supplementary Figure 3B). Training-induced associative plasticity in KCs became visible after the second training trial (trial 6 in Figure 1), one trial later than in DANs.

In the unpaired group, both DAN and KC responses to the CS decreased over trials in most compartments (Figure 4B and Supplementary Figures 3A,B). This response decrease could reflect non-associative plasticity due to repeated stimulus exposure, or it could be due to a decrease in CS concentration or bleaching of the calcium sensor (Supplementary Figure 4A). Indeed, additional control experiments revealed that DAN responses were sensitive to odorant concentration (100% compared to 60% of the initial odorant concentration): DANs generally responded stronger to the higher than to the lower odorant concentration (Supplementary Figure 5).

In both DANs and KCs, the responses to the control odorant MCH did not differ between the paired and unpaired group (Figure 4B and Supplementary Figures 3A,B), showing that the associative change in the DAN response was CS-specific and is not generalized to a different odor.

If *Drosophila* DANs were to encode the US prediction error similar to mammalian DANs (Schultz et al., 1997), then their US-induced responses should change in the course of the conditioning as the responses to the CS become stronger and the CS becomes predictive for the occurrence of the US. However, US-induced DAN (and KC) responses did not differ between the paired and unpaired group (Figure 4C, Supplementary Figures 1, 2, Supplementary Figure 3C,D). Moreover, unlike mammalian DANs, after conditioning *Drosophila* DANs did not change their activity when the US was omitted after presenting the CS (Figure 4A and Supplementary Figure 1). Therefore, the population of *TH-GAL4*-labeled DANs appears not to encode the US prediction error.

Taken together, associative plasticity differed between DANs and KCs in three ways: (1) Responses to the CS increased in DANs and decreased in KCs in the paired relative to the unpaired group. (2) Associative plasticity in the CS-induced responses occurred in DANs in eight out of nine compartments, whereas it occurred in KCs in three out of nine compartments (Figure 6A). (3) Associative plasticity in the response to the CS was visible after the 1st training trial in DANs and after the 2nd training trial in KCs. The lack of associative plasticity in US-induced responses suggests that DANs and KCs do not encode the US prediction error during trace conditioning.

### Associative plasticity of CS-induced spatial DAN activity patterns across MB compartments

It has been proposed that a conditioning-induced change in the cross-compartmental pattern of MBON activity induced by the CS reflects a change in the valence of the CS (Aso et al., 2014b; Owald and Waddell, 2015). Because DANs provide compartment-specific input to the MBs and drive the conditioning-induced changes of MBON responses (Cohn et al., 2015; Hige et al., 2015a), we asked whether and how odor—shock trace conditioning changes the cross-compartmental pattern of DAN activity (Figure 5A). To quantify the change in cross-compartmental activity patterns, we translated the recorded compartment responses for each fly into a vector that comprises the response strength of nine compartments. We quantified the dissimilarity between two activity patterns as the geometric angle between the two respective vectors in a 9-dimensional space. This yields a metric of pattern dissimilarity that is independent of response strength.

To analyze how the CS-induced spatial activity pattern changes during training, we compared the activity pattern for each trial with the pattern induced by the CS stimulation before the training (Figure 5B, “CS vs. 1st CS”). In both the paired and the unpaired group, the CS-induced activity patterns diverged from the initial pattern. This divergence was stronger in the unpaired than in the paired group (Figure 5B, “CS vs. 1st CS”). We next asked whether the US-induced activity patterns also change during conditioning. We therefore compared each US-induced activity pattern with the pattern induced by the first US stimulation (Figure 5B, “US vs. 1st US”). With repeated stimulation, the US-induced activity patterns diverged from the initial pattern. However, this divergence was less than in CS-induced activity patterns, and we found no difference in the effect between the paired and unpaired group (Figure 5B, “US vs. 1st US”). Thus, electric shock-induced activity patterns in DANs were unaffected by associative plasticity.

During odor—shock conditioning, the CS acquires the potential to elicit the conditioned response in *Drosophila* (Tully, 1984). Aversive conditioning experiments in *Drosophila* larvae revealed that the CS does not only become aversive, but that it actually gains predictive power (Schleyer et al., 2015). We therefore asked whether such an associative change in the predictive power of the CS could be reflected in the CS-induced spatial activity pattern of DANs. For example, does the CS-induced activity pattern become more similar to the US-induced activity pattern? To quantify whether the similarity between CS- and US-induced activity patterns changed during training, we compared each CS-induced activity pattern with the mean activity pattern induced by the US (Figure 5B, “CS vs. mean US”). In the paired group the mean angles between the CS- and US-induced activity patterns ranged between 0.6 and 1.0 rad (34–57°). In contrast, in the unpaired group the mean angles between the CS- and US-induced activity patterns diverged from 0.7 to 1.5 rad (40–86°). In the paired group the CS- and US-induced activity patterns were equally similar before and after training. However, in the unpaired group the CS- and US-induced activity patterns became less similar (Figure 5B, “CS vs. mean US”). In contrast to DANs, the cross-compartmental pattern of KC activity did not exhibit any associative changes (Supplementary Figure 6). In sum, associative plasticity preserved the degree of similarity between CS-induced and US-induced cross-compartmental activity patterns in DANs.

## Discussion

We investigated associative plasticity in the responses of DANs and their synaptic partners, the KCs, across the compartments of the *Drosophila* MB. Using calcium imaging, we recorded CS- and US-induced responses of a subpopulation of DANs (labeled by *TH-GAL4*) and of KCs (labeled by *OK107-GAL4*) during odor—shock trace conditioning (Galili et al., 2011). Note, that most compartments are innervated by multiple *TH-GAL4*-labeled DANs (Mao and Davis, 2009; Aso et al., 2010, 2014a). Therefore, the average activity that we recorded in most of the compartments might mask possible differences in the response properties and plasticity between individual DANs and KCs. Only DAN responses in the compartments γ2 and α'1 reflect the responses of a single neuron.

Across MB compartments, DANs and KCs differed in their response strength to odorants and electric shock (Figure 2), and they differed in CS-US pairing-induced plasticity (Figures 4, 6, Supplementary Figure 3). Compared to the unpaired control groups, KCs decreased their responses to the CS in all compartments of the β'-lobe and in the junction, while DANs increased their responses to the CS in all compartments of the γ- and β'-lobe, and in the junction. The occurrence of associative plasticity in DANs in the compartments γ3–5 and β'1 is surprising, given that these DANs are not known to be involved in odor—shock conditioning (Aso et al., 2010, 2012). Different to mammalian DANs (Schultz et al., 1997), after training there was neither an associative change in US-induced DAN responses nor a change of activity during US-omission after CS presentation. We therefore conclude, that *Drosophila* DANs do not encode the US-prediction error during classical conditioning.

### Compartment-specific responses to CS and US in DANs

Previous studies suggested that DANs in the MB lobes respond strongly to electric shock and weakly to odorants (Riemensperger et al., 2005; Mao and Davis, 2009). The compartment-resolved analysis of our calcium imaging data refines this picture: We confirm that DANs of all imaged compartments respond to both electric shock and odorants, and we show that their relative response strength to odorants and electric shock differs across compartments. For example, DANs innervating γ1 responded stronger to electric shock than to odorants, while DANs innervating β'2 responded equally strong to odorants and electric shock (Figure 2E). We found the strongest DAN responses to electric shock in the compartments γ1 and γ2. These compartments receive input from PPL1-γ1pedc and PPL1-γ2α'1 DANs that mediate electric shock reinforcement (Aso et al., 2010, 2012). In all compartments, except in α1/α'1, the DAN response strength correlated positively with the current strength encountered by individual flies (Figure 3B). Thus, DANs are capable to encode the strength of the electric shock US (Mao and Davis, 2009), and this property may account for the positive dependence between electric shock strength and learning performance in flies (Tully and Quinn, 1985).

### Compartment-specific responses to CS and US in KCs

Calcium responses in KCs differ between MB lobes (Turner et al., 2008; Lin A. C. et al., 2014), and they differ between the compartments of a given lobe, possibly due to compartment-specific modulation by DANs and MBONs (Tanaka et al., 2008; Aso et al., 2014a; Cohn et al., 2015). KCs in γ2 and γ3 responded strongest to odorants (Figure 2A and Supplementary Figure 2), confirming the results of Cohn et al. (2015). KCs generally responded only weakly to electric shocks. Previously published strong KC responses to electric shock may be because electric shocks were applied to the flies' abdomen rather than to their legs, which might have resulted in a stronger stimulation (Akalal et al., 2010).

### Associative plasticity in DAN and KC responses

The associative strengthening of DAN responses to the olfactory CS (as compared to the unpaired control group; Figure 6A), confirms the previous report by Riemensperger et al. (2005). Associative plasticity occurred in those DANs that innervate the MBs (PPL1 and PAM cluster DANs; note that the used *TH-GAL4* driver line covers only a small subpopulation of PAM neurons (Aso et al., 2012; Pech et al., 2013) but not in DANs that innervate the central complex (PPL1 and PPM3 cluster DANs). This is in line with the established role of MB innervating-DANs in associative memory formation, while central complex-innervating DANs are involved in behaviors such as locomotion (Kong et al., 2010), wakefulness (Liu et al., 2012), arousal (Ueno et al., 2012), and aggression (Alekseyenko et al., 2013), and are therefore not expected to show odor—shock conditioning-induced plasticity.

In contrast to previous studies (Wang et al., 2008; Akalal et al., 2010, 2011), we did not find an associative increase in KC calcium responses to the CS in the MB-lobes after odor—shock conditioning (Figure 6A). This may indicate either a difference between trace conditioning (this study) and standard conditioning (published data), or a difference in other experimental parameters that may also account for inconsistencies in the published effects of odor—shock conditioning (Zhang and Roman, 2013; Boto et al., 2014; Hige et al., 2015a).

The associative decrease in KC responses in the β'-lobe compartments (Figure 4B) is in line with previous studies that showed conditioning-induced depression of KC-to-MBON synapses (Cohn et al., 2015; Hige et al., 2015a). Therefore, we propose that the associative decrease in KC responses to the CS reflects a presynaptic depression at KC-to-MBON synapses in β'-lobe compartments (Figure 6B).

What is the site of neuronal plasticity that underlies the relative increase in DANs' responses to the olfactory CS? Riemensperger et al. (2005) proposed that DANs get odorant-driven excitatory input via a MBON feedback loop that is strengthened during odor—shock conditioning. However, the DAN population is composed of different neuron types that, to our knowledge, do not share a common input neither from MBONs nor from other neurons that could explain the global associative plasticity across MB compartments. Because KCs presumably provide the only common odor-driven input to all MB-innervating DANs, we suggest that the site of associative plasticity is located in a KC-to-DAN synapse. Indeed, KC-to-DAN synapses have recently been reported in *Drosophila* (Cervantes-Sandoval et al., 2017). Associative increase in CS-induced DAN responses occurred despite unaltered or decreased KC responses in the same compartment. This suggests that the associative plasticity occurs post-synaptic in DANs and is not inherited from KCs (Figure 6B). Note, that the associative changes in DANs' response strength could be influenced by lateral modulation via other compartments, as has been shown in the study of Cohn et al. (2015).

### Neuronal substrate of sensory odor traces

What is the neuronal substrate of CS-US coincidence detection in DANs and KCs? *Drosophila* trace conditioning depends on dopamine receptor-triggered signaling in KCs (Shuai et al., 2011), as is the case for standard conditioning (Kim et al., 2007; Qin et al., 2012). However, the CS-US coincidence detection mechanism in trace conditioning is unknown (Galili et al., 2011; Shuai et al., 2011; Dylla et al., 2013). In standard conditioning the CS-induced increase in KCs' calcium concentration coincides with the US-(dopamine)-induced second messengers, which is thought to synergistically activate the rutabaga adenylyl cyclase (Duerr and Quinn, 1982; Dudaí et al., 1983; Tomchik and Davis, 2009; Gervasi et al., 2010), and ultimately alters the strength of KC-to-MBON synapses (Dubnau et al., 2001; McGuire et al., 2001; Schwaerzel et al., 2003; Séjourné et al., 2011; Pai et al., 2013; Zhang and Roman, 2013; Aso et al., 2014b; Bouzaiane et al., 2015; Cohn et al., 2015; Hige et al., 2015a; Owald et al., 2015). This mechanism would not work for trace conditioning, because (1) at the time the US occurs, CS-induced increase in KCs' calcium concentration is back to baseline levels (Figure 2D, Supplementary Figure 2), and (2) trace conditioning does not involve the rutabaga adenylyl cyclase (Shuai et al., 2011). We therefore hypothesize that a non-rutabaga adenylyl cyclase (Adams et al., 2000) or a protein kinase C (Choi et al., 1991; Widmann et al., 2016) could serve as a molecular coincidence detector for the CS trace and the US. For example, the CS-induced calcium and dopamine signaling could lead to a sustained activation of an adenylyl cyclase or protein kinase C in KCs, which then would increase synergistically and drive synaptic plasticity during the US-induced dopamine signaling.

### Function of associative plasticity in CS-induced responses in DANs

DAN responses to odorants and associative strengthening of DAN responses to the CS-odorant are not included in current models of associative learning in the MB (Busto et al., 2010; Owald and Waddell, 2015). However, associative plasticity is a common feature of US-mediating neurons, which occurs in mammalian and *Drosophila* DANs (Schultz et al., 1997; Riemensperger et al., 2005), and in an octopaminergic neuron in honey bees (Hammer, 1993).

What could be the function of odorant-induced responses and odor—shock conditioning-induced plasticity in DANs? MB-innervating DANs strengthened their response to the CS (as compared to the unpaired group) during odor—shock conditioning (Figure 4B), in line with Riemensperger et al. (2005). However, other than in monkey DANs (Montague et al., 1996; Schultz et al., 1997; Steinberg et al., 2013), we did not observe associative plasticity in DANs' response to the US (Figure 4C). Our data therefore support the idea that *Drosophila* DANs encode predictive power of the CS, e.g., US-prediction, but not the US-prediction error during classical conditioning (Riemensperger et al., 2005).

We found shock-induced responses and associative plasticity in DANs that are not involved in odor—shock conditioning, for example in DANs innervating β'1, γ3, γ4, and γ5 (Aso et al., 2010, 2012). This suggests that those DANs serve a function in aversive odor learning which is not captured by the commonly applied conditioning paradigms. For example, the relative strengthening of CS-induced responses could mediate reinforcement during second-order conditioning, in which a previously reinforced CS_1_ can act as US in subsequent conditioning of a second CS_2_ (Pavlov, 1927). As *Drosophila* is capable of second-order learning (Brembs and Heisenberg, 2001; Tabone and de Belle, 2011), this theory can be tested in behavioral experiments: if associative strengthening of DAN responses to the CS underlies CS_1_-induced reinforcement in second-order conditioning, then preventing associative plasticity in DANs, or blocking their output during CS_2_–CS_1_ pairing should abolish second-order conditioning.

The occurrence of CS-induced responses and associative plasticity in most of the MB-innervating DANs suggests that the separation between the CS- and US-pathway and between different US-pathways is less strict than suggested in current models of associative learning in the MB. Associative plasticity in the spatial pattern of CS-induced DAN responses (Figure 5) makes them a potential neuronal substrate for encoding the US identity (Galili et al., 2011; Burke et al., 2012; Das et al., 2014; Lin S. et al., 2014; Cohn et al., 2015; Huetteroth et al., 2015) in CS-US memories and the predictive power of a CS.

Our data revealed similar response properties and plasticity rules across *Drosophila* DANs in the γ- and β'-lobe. This contrasts with their anatomical (Tanaka et al., 2008; Mao and Davis, 2009; Aso et al., 2014a) and functional heterogeneity (Krashes et al., 2009; Aso et al., 2010, 2012; Galili et al., 2011; Berry et al., 2012; Burke et al., 2012; Plaçais et al., 2012; Das et al., 2014; Cohn et al., 2015; Aso and Rubin, 2016; Felsenberg et al., 2017), which indicates yet undiscovered mechanisms and functions of DAN plasticity. Note, that we could not test whether the flies learned in the imaging setup, as currently no behavioral readout exists for odor—shock conditioning during physiological experiments. Nevertheless, since we used a conditioning protocol and stimulus application comparable to an established behavioral paradigm, we believe that the associative plasticity in neuronal responses that we found underlies behavioral associative plasticity. Therewith our data lay the foundations for causal studies on the function of associative plasticity in DANs.

## Author contributions

KD conducted all the experiments and analyzed the data, except for the experiments in Supplementary Figure 5 which were done by GR. KD, GR, CG, and PS designed the experiments and wrote the paper.

### Conflict of interest statement

The authors declare that the research was conducted in the absence of any commercial or financial relationships that could be construed as a potential conflict of interest.

## References

[B1] Adams M. D. Celniker S. E. Holt R. A. Evans C. A. Gocayne J. D. Amanatides P. G.. (2000). The genome sequence of *Drosophila Melanogaster*. Science 287, 2185–2195. 10.1126/science.287.5461.2185 10731132

[B2] Akalal D. B. G. Yu D. Davis R. L. (2010). A late-phase, long-term memory trace forms in the γ neurons of Drosophila mushroom bodies after olfactory classical conditioning. J. Neurosci. 30, 16699–16708. 10.1523/JNEUROSCI.1882-10.2010 21148009 PMC3380342

[B3] Akalal D. B. G. Yu D. Davis R. L. (2011). The long-term memory trace formed in the Drosophila α/β mushroom body neurons is abolished in long-term memory mutants. J. Neurosci. 31, 5643–5647. 10.1523/JNEUROSCI.3190-10.2011 21490205 PMC3118425

[B4] Alekseyenko O. V. Chan Y. B. Li R. Kravitz E. A. (2013). Single dopaminergic neurons that modulate aggression in Drosophila. Proc. Natl. Acad. Sci. U.S.A. 110, 6151–6156. 10.1073/pnas.1303446110 23530210 PMC3625311

[B5] Aso Y. Rubin G. M. (2016). Dopaminergic neurons write and update memories with cell-type-specific rules. Elife 5, 1–15. 10.7554/eLife.16135 27441388 PMC4987137

[B6] Aso Y. Hattori D. Yu Y. Johnston R. M. Iyer N. A. Ngo T. T.. (2014a). The neuronal architecture of the mushroom body provides a logic for associative learning. Elife 3:e04577. 10.7554/elife.04577 25535793 PMC4273437

[B7] Aso Y. Herb A. Ogueta M. Siwanowicz I. Templier T. Friedrich A. B.. (2012). Three dopamine pathways induce aversive odor memories with different stability. PLoS Genet. 8:e1002768. 10.1371/journal.pgen.1002768 22807684 PMC3395599

[B8] Aso Y. Sitaraman D. Ichinose T. Kaun K. R. Vogt K. Belliart-Guérin G.. (2014b). Mushroom body output neurons encode valence and guide memory-based action selection in Drosophila. Elife 3:e04580. 10.7554/eLife.04580 25535794 PMC4273436

[B9] Aso Y. Siwanowicz I. Bräcker L. Ito K. Kitamoto T. Tanimoto H. (2010). Specific dopaminergic neurons for the formation of labile aversive memory. Curr. Biol. 20, 1445–1451. 10.1016/j.cub.2010.06.048 20637624 PMC2929706

[B10] Berry J. A. Cervantes-Sandoval I. Chakraborty M. Davis R. L. (2015). Sleep facilitates memory by blocking dopamine neuron-mediated forgetting. Cell 161, 1656–1667. 10.1016/j.cell.2015.05.027 26073942 PMC4671826

[B11] Berry J. A. Cervantes-Sandoval I. Nicholas E. P. Davis R. L. (2012). Dopamine is required for learning and forgetting in Drosophila. Neuron 74, 530–542. 10.1016/j.neuron.2012.04.007 22578504 PMC4083655

[B12] Boto T. Louis T. Jindachomthong K. Jalink K. Tomchik S. M. (2014). Dopaminergic modulation of cAMP drives nonlinear plasticity across the Drosophila mushroom body lobes. Curr. Biol. 24, 822–831. 10.1016/j.cub.2014.03.021 24684937 PMC4019670

[B13] Bouzaiane E. Trannoy S. Scheunemann L. Plaçais P. Y. Preat T. (2015). Two independent mushroom body output circuits retrieve the six discrete components of Drosophila aversive memory. Cell Rep. 11, 1280–1292. 10.1016/j.celrep.2015.04.044 25981036

[B14] Brembs B. Heisenberg M. (2001). Conditioning with compound stimuli in *Drosophila Melanogaster* in the flight simulator. J. Exp. Biol. 204(Pt 16), 2849–2859. 11683440 10.1242/jeb.204.16.2849

[B15] Burke C. J. Huetteroth W. Owald D. Perisse E. Krashes M. J. Das G.. (2012). Layered reward signalling through octopamine and dopamine in Drosophila. Nature 492, 433–437. 10.1038/nature11614 23103875 PMC3528794

[B16] Busto G. U. Cervantes-Sandoval I. Davis R. L. (2010). Olfactory learning in Drosophila. Physiology 25, 338–346. 10.1152/physiol.00026.2010 21186278 PMC3380424

[B17] Cervantes-Sandoval I. Phan A. Chakraborty M. Davis R. L. (2017). Reciprocal synapses between mushroom body and dopamine neurons form a positive feedback loop required for learning. Elife 6:e23789. 10.7554/elife.23789 28489528 PMC5425253

[B18] Choi K. W. Smith R. F. Buratowski R. M. Quinn W. G. (1991). Deficient protein kinase C activity in turnip, a Drosophila learning mutant. J. Biol. Chem. 266, 15999–15606. 1874743

[B19] Cohn R. Morantte I. Ruta V. (2015). Coordinated and compartmentalized neuromodulation shapes sensory processing in Drosophila. Cell 163, 1742–1755. 10.1016/j.cell.2015.11.019 26687359 PMC4732734

[B20] Connolly J. B. Roberts I. J. Armstrong J. D. Kaiser K. Forte M. Tully T.. (1996). Associative learning disrupted by impaired Gs signaling in Drosophila mushroom bodies. Science 274, 2104–2107. 10.1126/science.274.5295.2104 8953046

[B21] Das G. Klappenbach M. Vrontou E. Perisse E. Clark C. M. Burke C. J.. (2014). Drosophila learn opposing components of a compound food stimulus. Curr. Biol. 24, 1723–1730. 10.1016/j.cub.2014.05.078 25042590 PMC4131107

[B22] Dubnau J. Grady L. Kitamoto T. Tully T. (2001). Disruption of neurotransmission in Drosophila mushroom body blocks retrieval but not acquisition of memory. Nature 411, 476–480. 10.1038/35078077 11373680

[B23] Dudaí Y. Uzzan A. Zvi S. (1983). Abnormal activity of adenylate cyclase in the Drosophila memory mutant rutabaga. Neurosci. Lett. 42, 207–212. 10.1016/0304-3940(83)90408-1 6420732

[B24] Duerr J. S. Quinn W. G. (1982). Three Drosophila mutations that block associative learning also affect habituation and sensitization. Proc. Natl. Acad. Sci. U.S.A. 79, 3646–3650. 10.1073/pnas.79.11.3646 6808513 PMC346480

[B25] Dylla K. V. Galili D. S. Szyszka P. Lüdke A. (2013). Trace conditioning in insects-keep the trace! Front. Physiol. 4:67. 10.3389/fphys.2013.00067 23986710 PMC3750952

[B26] Felsenberg J. Barnstedt O. Cognigni P. Lin S. Waddell S. (2017). Re-evaluation of learned information in Drosophila. Nature 544, 240–244. 10.1038/nature21716 28379939 PMC5392358

[B27] Friggi-Grelin F. Coulom H. Meller M. Gomez D. Hirsh J. Birman S. (2003). Targeted gene expression in Drosophila dopaminergic cells using regulatory sequences from tyrosine hydroxylase. J. Neurobiol. 54, 618–627. 10.1002/neu.10185 12555273

[B28] Galili D. S. Lüdke A. Galizia C. G. Szyszka P. Tanimoto H. (2011). Olfactory trace conditioning in Drosophila. J. Neurosci. 31, 7240–7248. 10.1523/JNEUROSCI.6667-10.2011 21593308 PMC6622595

[B29] Gerber B. Ullrich J. (1999). No evidence for olfactory blocking in honeybee classical conditioning. J. Exp. Biol. 202(Pt 13), 1839–1854. 10359686 10.1242/jeb.202.13.1839

[B30] Gervasi N. Tchénio P. Preat T. (2010). PKA Dynamics in a Drosophila learning center: coincidence detection by rutabaga adenylyl cyclase and spatial regulation by dunce phosphodiesterase. Neuron 65, 516–529. 10.1016/j.neuron.2010.01.014 20188656

[B31] Guerrieri F. Lachnit H. Gerber B. Giurfa M. (2005). Olfactory blocking and odorant similarity in the honeybee. Learn. Mem. 12, 86–95. 10.1101/lm.79305 15805307 PMC1074325

[B32] Hammer M. (1993). An indentified neuron mediates the unconditioned stimulus in associative olfactory learning in honeybees. Nature 366, 59–63. 10.1038/366059a0 24308080

[B33] Hige T. Aso Y. Modi M. N. Rubin G. M. Turner G. C. (2015a). Heterosynaptic plasticity underlies aversive olfactory learning in Drosophila. Neuron 88, 985–998. 10.1016/j.neuron.2015.11.003 26637800 PMC4674068

[B34] Hige T. Aso Y. Rubin G. M. Turner G. C. (2015b). Plasticity-driven individualization of olfactory coding in mushroom body output neurons. Nature 526, 258–262. 10.1038/nature15396 26416731 PMC4860018

[B35] Hosler J. S. Smith B. H. (2000). Blocking and the detection of odor components in blends. J. Exp. Biol. 203(Pt 18), 2797–2806. 10952879 10.1242/jeb.203.18.2797

[B36] Huetteroth W. Perisse E. Lin S. Klappenbach M. Burke C. Waddell S. (2015). Sweet taste and nutrient value subdivide rewarding dopaminergic neurons in Drosophila. Curr. Biol. 25, 751–758. 10.1016/j.cub.2015.01.036 25728694 PMC4372253

[B37] Kamin L. J. (1969). Predictability, Surprise, Attention and Conditioning, in Punishment and Aversive Behavior, eds Campbell B. A. Church R. M. (New York, NY: Appleton-Century-Crofts), 279–296.

[B38] Kim Y. C. Lee H. G. Han K. A. (2007). D1 dopamine receptor dDA1 is required in the mushroom body neurons for aversive and appetitive learning in Drosophila. J. Neurosci. 27, 7640–7647. 10.1523/JNEUROSCI.1167-07.2007 17634358 PMC6672866

[B39] Kong E. C. Woo K. Li H. Lebestky T. Mayer N. Sniffen M. R.. (2010). A pair of dopamine neurons target the D1-like dopamine receptor DopR in the central complex to promote ethanol-stimulated locomotion in Drosophila. PLoS ONE 5:e9954. 10.1371/journal.pone.0009954 20376353 PMC2848596

[B40] Krashes M. J. DasGupta S. Vreede A. White B. Armstrong J. D. Waddell S. (2009). A neural circuit mechanism integrating motivational state with memory expression in Drosophila. Cell 139, 416–427. 10.1016/j.cell.2009.08.035 19837040 PMC2780032

[B41] Lewis L. P. Siju K. P. Aso Y. Friedrich A. B. Bulteel A. J. Rubin G. M.. (2015). A higher brain circuit for immediate integration of conflicting sensory information in Drosophila. Curr. Biol. 25, 2203–2214. 10.1016/j.cub.2015.07.015 26299514

[B42] Lin A. C. Bygrave A. M. de Calignon A. Lee T. Miesenböck G. (2014). Sparse, decorrelated odor coding in the mushroom body enhances learned odor discrimination. Nat. Neurosci. 17, 559–568. 10.1038/nn.3660 24561998 PMC4000970

[B43] Lin S. Owald D. Chandra V. Talbot C. Huetteroth W. Waddell S. (2014). Neural correlates of water reward in thirsty Drosophila. Nat. Neurosci. 17, 1536–1542. 10.1038/nn.3827 25262493 PMC4213141

[B44] Liu C. Plaçais P. Y. Yamagata N. Pfeiffer B. D. Aso Y. Friedrich A. B.. (2012). A subset of dopamine neurons signals reward for odour memory in Drosophila. Nature 488, 512–516. 10.1038/nature11304 22810589

[B45] Mao Z. Davis R. L. (2009). Eight different types of dopaminergic neurons innervate the Drosophila mushroom body neuropil: anatomical and physiological heterogeneity. Front. Neural Circ. 3:5. 10.3389/neuro.04.005.2009 19597562 PMC2708966

[B46] Masek P. Worden K. Aso Y. Rubin G. M. Keene A. C. (2015). A dopamine-modulated neural circuit regulating aversive taste memory in Drosophila. Curr. Biol. 25, 1535–1541. 10.1016/j.cub.2015.04.027 25981787 PMC4873318

[B47] McGuire S. E. Le P. T. Davis R. L. (2001). The role of Drosophila mushroom body signaling in olfactory memory. Science 293, 1330–1333. 10.1126/science.1062622 11397912

[B48] Montague P. R. Dayan P. Sejnowski T. J. (1996). A framework for mesencephalic dopamine systems based on predictive hebbian learning. J. Neurosci. 16, 1936–1947. 8774460 10.1523/JNEUROSCI.16-05-01936.1996 PMC6578666

[B49] Murthy M. Fiete I. Laurent G. (2008). Testing odor response stereotypy in the Drosophila mushroom body. Neuron 59, 1009–1023. 10.1016/j.neuron.2008.07.040 18817738 PMC2654402

[B50] Musso P. Y. Tchenio P. Preat T. (2015). Delayed dopamine signaling of energy level builds appetitive long-term memory in Drosophila. Cell Rep. 10, 1023–1031. 10.1016/j.celrep.2015.01.036 25704807

[B51] Nall A. H. Shakhmantsir I. Cichewicz K. Birman S. Hirsh J. Sehgal A. (2016). Caffeine promotes wakefulness via dopamine signaling in Drosophila. Sci. Rep. 6:20938. 10.1038/srep20938 26868675 PMC4751479

[B52] Owald D. Waddell S. (2015). Olfactory learning skews mushroom body output pathways to steer behavioral choice in Drosophila. Curr. Opin. Neurobiol. 35, 178–184. 10.1016/j.conb.2015.10.002 26496148 PMC4835525

[B53] Owald D. Felsenberg J. Talbot C. B. B. Das G. Perisse E. Huetteroth W.. (2015). Activity of defined mushroom body output neurons underlies learned olfactory behavior in Drosophila. Neuron 86, 417–427. 10.1016/j.neuron.2015.03.025 25864636 PMC4416108

[B54] Pai T. P. Chen C. C. Lin H. H. Chin A. L. Lai J. S. Lee P. T.. (2013). Drosophila ORB protein in two mushroom body output neurons is necessary for long-term memory formation. Proc. Natl. Acad. Sci. U.S.A. 110, 7898–7903. 10.1073/pnas.1216336110 23610406 PMC3651462

[B55] Pavlov P. I. (1927). Conditioned Reflexes: An Investigation of the Physiological Activity of the Cerebral Cortex. London: Oxford University Press. 10.5214/ans.0972-7531.1017309 PMC4116985 25205891

[B56] Pech U. Pooryasin A. Birman S. Fiala A. (2013). Localization of the contacts between Kenyon cells and aminergic neurons in the *Drosophila Melanogaster* brain using splitGFP reconstitution. J. Comp. Neurol. 521, 3992–4026. 10.1002/cne.23388 23784863

[B57] Pitman J. L. DasGupta S. Krashes M. J. Leung B. Perrat P. N. Waddell S. (2017). There are many ways to train a fly. Fly 3, 3–9. 10.4161/fly.3.1.7726 19164943 PMC2814444

[B58] Plaçais P. Y. Trannoy S. Friedrich A. B. Tanimoto H. Preat T. (2013). Two pairs of mushroom body efferent neurons are required for appetitive long-term memory retrieval in Drosophila. Cell Rep. 5, 769–780. 10.1016/j.celrep.2013.09.032 24209748

[B59] Plaçais P. Y. Trannoy S. Isabel G. Aso Y. Siwanowicz I. Belliart-Guérin G.. (2012). Slow oscillations in two pairs of dopaminergic neurons gate long-term memory formation in Drosophila. Nat. Neurosci. 15, 592–599. 10.1038/nn.3055 22366756

[B60] Qin H. Cressy M. Li W. Coravos J. S. Izzi S. A. Dubnau J. (2012). Gamma neurons mediate dopaminergic input during aversive olfactory memory formation in Drosophila. Curr. Biol. 22, 608–614. 10.1016/j.cub.2012.02.014 22425153 PMC3326180

[B61] Quinn W. G. Harris W. A. Benzer S. (1974). Conditioned behavior in *Drosophila Melanogaster*. Proc. Natl. Acad. Sci. U.S.A. 71, 708–712. 10.1073/pnas.71.3.708 4207071 PMC388082

[B62] R Core Team (2014). A Language and Environment for Statistical Computing. Available online at: http://www.r-project.org/

[B63] Rescorla R. A. Wagner A. R. (1972). A theory of pavlovian conditioning: variations in the effectiveness of reinforcement and nonreinforcement. Class. Condition. II Curr. Res. Theor. 21, 64–99.

[B64] Riemensperger T. Völler T. Stock P. Buchner E. Fiala A. (2005). Punishment prediction by dopaminergic neurons in Drosophila. Curr. Biol. 15, 1953–1960. 10.1016/j.cub.2005.09.042 16271874

[B65] Schleyer M. Miura D. Tanimura T. Gerber B. (2015). Learning the specific quality of taste reinforcement in larval Drosophila. Elife 2015, 1–10. 10.7554/elife.04711 PMC4302267 25622533

[B66] Schultz W. (2013). Updating dopamine reward signals. Curr. Opin. Neurobiol. 23, 229–238. 10.1016/j.conb.2012.11.012 23267662 PMC3866681

[B67] Schultz W. Apicella P. Ljungberg T. (1993). Responses of monkey dopamine neurons to reward and conditioned stimuli during successive steps of learning a delayed response task. J. Neurosci. 13, 900–913. 8441015 10.1523/JNEUROSCI.13-03-00900.1993 PMC6576600

[B68] Schultz W. Dayan P. Montague P. R. (1997). A neural substrate of prediction and reward. Science 275, 1593–1599. 10.1126/science.275.5306.1593 9054347

[B69] Schwaerzel M. Monastirioti M. Scholz H. Friggi-Grelin F. Birman S. Heisenberg M. (2003). Dopamine and octopamine differentiate between aversive and appetitive olfactory memories in Drosophila. J. Neurosci. 23, 10495–10502. 14627633 10.1523/JNEUROSCI.23-33-10495.2003 PMC6740930

[B70] Séjourné J. Plaçais P. Y. Aso Y. Siwanowicz I. Trannoy S. Thoma V.. (2011). Mushroom body efferent neurons responsible for aversive olfactory memory retrieval in Drosophila. Nat. Neurosci. 14, 903–910. 10.1038/nn.2846 21685917

[B71] Shuai Y. Hu Y. Qin H. Campbell R. A. Zhong Y. (2011). Distinct molecular underpinnings of Drosophila olfactory trace conditioning. Proc. Natl. Acad. Sci. U.S.A. 108, 20201–20206. 10.1073/pnas.1107489109 22123966 PMC3250181

[B72] Sitaraman D. Aso Y. Rubin G. M. Nitabach M. N. (2015). Control of sleep by dopaminergic inputs to the Drosophila mushroom body. Front. Neural Circuits 9:73 10.3389/fncir.2015.00073 26617493 PMC4637407

[B73] Smith B. H. Cobey S. (1994). The olfactory memory of the honeybee *Apis mellifera*. II. Blocking between odorants in binary mixtures. J. Exp. Biol. 195, 91–108. 7964421 10.1242/jeb.195.1.91

[B74] Steinberg E. E. Keiflin R. Boivin J. R. Witten I. B. Deisseroth K. Janak P. H. (2013). A causal link between prediction errors, dopamine neurons and learning. Nat. Neurosci. 16, 966–973. 10.1038/nn.3413 23708143 PMC3705924

[B75] Szyszka P. Demmler C. Oemisch M. Sommer L. Biergans S. Birnbach B.. (2011). Mind the gap: olfactory trace conditioning in honeybees. J. Neurosci. 31, 7229–7239. 10.1523/JNEUROSCI.6668-10.2011 21593307 PMC6622586

[B76] Tabone C. J. de Belle J. S. (2011). Second-order conditioning in Drosophila. Learn. Mem. 18, 250–253. 10.1101/lm.2035411 21441302 PMC3072777

[B77] Tanaka N. K. Tanimoto H. Ito K. (2008). Neuronal assemblies of the Drosophila mushroom body. J. Comp. Neurol. 508, 711–755. 10.1002/cne.21692 18395827

[B78] Terao K. Matsumoto Y. Mizunami M. (2015). Critical evidence for the prediction error theory in associative learning. Sci. Rep. 5:8929. 10.1038/srep08929 25754125 PMC4354000

[B79] Tian L. Hires S. A. Mao T. Huber D. Chiappe M. E. Chalasani S. H.. (2009). Imaging neural activity in worms, flies and mice with improved GCaMP calcium indicators. Nat. Methods 6, 875–881. 10.1038/nmeth.1398 19898485 PMC2858873

[B80] Tomchik S. M. Davis R. L. (2009). Dynamics of learning-related cAMP signaling and stimulus integration in the Drosophila olfactory pathway. Neuron 64, 510–521. 10.1016/j.neuron.2009.09.029 19945393 PMC4080329

[B81] Tully T. (1984). Drosophila learning: behavior and biochemistry. Behav. Genet. 14, 527–557. 10.1007/BF01065446 6395853

[B82] Tully T. Quinn W. G. (1985). Classical conditioning and retention in normal and mutant *Drosophila Melanogaster. J. Comp. Physiology*. A 157, 263–277. 10.1007/BF01350033 3939242

[B83] Turner G. C. Bazhenov M. Laurent G. (2008). Olfactory representations by Drosophila mushroom body neurons. J. Neurophysiol. 99, 734–746. 10.1152/jn.01283.2007 18094099

[B84] Ueno T. Tomita J. Tanimoto H. Endo K. Ito K. Kume S.. (2012). Identification of a dopamine pathway that regulates sleep and arousal in Drosophila. Nat. Neurosci. 15, 1516–1523. 10.1038/nn.3238 23064381

[B85] Waddell S. (2013). Reinforcement signalling in Drosophila; dopamine does it all after all. Curr. Opin. Neurobiol. 23, 324–329. 10.1016/j.conb.2013.01.005 23391527 PMC3887340

[B86] Wang Y. Mamiya A. Chiang A. S. Zhong Y. (2008). Imaging of an early memory trace in the Drosophila mushroom body. J. Neurosci. 28, 4368–4376. 10.1523/JNEUROSCI.2958-07.2008 18434515 PMC3413309

[B87] Widmann A. Artinger M. Biesinger L. Boepple K. Peters C. Schlechter J.. (2016). Genetic dissection of aversive associative olfactory learning and memory in Drosophila larvae. PLoS Genet. 12:e1006378. 10.1371/journal.pgen.1006378 27768692 PMC5074598

[B88] Zhang S. Roman G. (2013). Presynaptic inhibition of gamma lobe neurons is required for olfactory learning in Drosophila. Curr. Biol. 23, 2519–2527. 10.1016/j.cub.2013.10.043 24291093

