# Supplementary material for: Trace Conditioning in Drosophila Induces Associative Plasticity in Mushroom Body Kenyon Cells and Dopaminergic Neurons
Source: Front Neural Circuits. 2017 Jun 20;11:42. doi: 10.3389/fncir.2017.00042 (PMC5476701; doi:10.3389/fncir.2017.00042)
Supplement: Supplementary file 1 [file Table1.pdf]

Supplementary Material

Trace conditioning in *Drosophila* induces associative plasticity in mushroom body Kenyon cells and dopaminergic neurons

Kristina V. Dylla[1], Georg Raiser[1], C. Giovanni Galizia[1], Paul Szyszka[1*]

[1] University of Konstanz, Department of Biology, Neurobiology, 78457 Konstanz, Germany

***Correspondence:**

Paul Szyszka

paul.szyszka@uni-konstanz.de

Supplementary Table 1: Results of the ANOVAs run on linear mixed-effect models.

Figure	Model	Compartment	Factor	df1	df2	F	p
NA (hemispheres: KCs, paired, shock)	difference in response strength ~ hemisphere, random = ~1|fly/compartment/trial	all	(Intercept)	1	201	2.017	0.1571
			hemisphere	1	201	3.479	0.0636
NA (hemispheres: KCs, unpaired, shock)		all	(Intercept)	1	217	13.966	0.0002
			hemisphere	1	217	3.077	0.0808
NA (hemispheres: DANs, paired, shock)		all	(Intercept)	1	315	1805.556	<.0001
			hemisphere	1	315	1.444	0.2304
NA (hemispheres: DANs, unpaired, shock)		all	(Intercept)	1	331	1257.702	<.0001
			hemisphere	1	331	4.718	0.0306
NA (hemispheres: KCs, paired, CS)		all	(Intercept)	1	403	55.757	<.0001
			hemisphere	1	403	24.21	<.0001
NA (hemispheres: KCs, unpaired, CS)		all	(Intercept)	1	435	27.58	<.0001
			hemisphere	1	435	1.42	0.2341
NA (hemispheres: DANs, paired, CS)		all	(Intercept)	1	631	0.256	0.6133
			hemisphere	1	631	3.816	0.0512
NA (hemispheres: DANs, unpaired, CS)		all	(Intercept)	1	663	42.795	<.0001
			hemisphere	1	663	0.848	0.3574
Figure 4B (DANs) and Suppl. Figure 3A	training-induced difference in response strength ~ trial*compartment*protocol, random = ~1|fly	all	(Intercept)	1	1332	9.545	0.002
			protocol	1	45	17.423	0.0001
			trial	1	1332	36.507	<.0001
			compartment	11	383	7.781	<.0001
			protocol:trial	1	1332	47.101	<.0001

			protocol:compartment	11	383	3.988	<.0001
			trial:compartment	11	1332	2.027	0.0229
			protocol:trial:compartment	11	1332	1.261	0.2418
	training-induced difference in response strength ~ trial*protocol, random = ~1|fly	γ1	(Intercept)	1	121	3.334	0.0703
			protocol	1	39	20.173	0.0001
			trial	1	121	0.629	0.4293
			protocol:trial	1	121	8.181	0.005
		γ2	(Intercept)	1	139	18.897	<.0001
			protocol	1	45	6.901	0.0117
			trial	1	139	9.198	0.0029
			protocol:trial	1	139	9.386	0.0026
		γ3	(Intercept)	1	133	3.211	0.0754
			protocol	1	43	6.438	0.0149
			trial	1	133	1.518	0.2201
			protocol:trial	1	133	7.395	0.0074
		γ4	(Intercept)	1	133	0.01	0.919
			protocol	1	43	18.432	0.0001
			trial	1	133	0.63	0.4286
			protocol:trial	1	133	8.579	0.004
		γ5	(Intercept)	1	139	0.284	0.5947
			protocol	1	45	20.412	<.0001
			trial	1	139	1.349	0.2474
			protocol:trial	1	139	7.628	0.0065
		β'1	(Intercept)	1	109	14.659	0.0002
			protocol	1	35	5.592	0.0237
			trial	1	109	37.768	<.0001
			protocol:trial	1	109	1.745	0.1893
		β'2	(Intercept)	1	139	28.955	<.0001
			protocol	1	45	33.183	<.0001
			trial	1	139	4.3	0.04
			protocol:trial	1	139	11.866	0.0008
		junction	(Intercept)	1	115	1.158	0.2841
			protocol	1	37	19.272	0.0001
			trial	1	115	1.461	0.2293
			protocol:trial	1	115	6.744	0.0106
		α1/α'1	(Intercept)	1	25	0.338	0.5664
			protocol	1	7	2.658	0.147
			trial	1	25	0.004	0.9512
			protocol:trial	1	25	1.014	0.3235
		FB1	(Intercept)	1	109	0.328	0.5682
			protocol	1	35	0.109	0.7429
			trial	1	109	2.152	0.1453
			protocol:trial	1	109	1.344	0.2489

		FB2	(Intercept)	1	97	1.134	0.2896
			protocol	1	31	0.184	0.6708
			trial	1	97	4.655	0.0334
			protocol:trial	1	97	1.74	0.1903
		EB	(Intercept)	1	73	0.529	0.4692
			protocol	1	23	0.004	0.9531
			trial	1	73	3.332	0.072
			protocol:trial	1	73	0.194	0.6612
Figure 4C and Suppl. Figure 3C	training-induced difference in response strength ~ trial*compartment*protocol, random = ~1|fly	all	(Intercept)	1	428	2441.799	<.0001
			protocol	1	45	3.174	0.0816
			trial	1	428	2.908	0.0889
			compartment	11	383	12.06	<.0001
			protocol:trial	1	428	0.03	0.8637
			protocol:compartment	11	383	0.939	0.5025
			trial:compartment	11	428	0.37	0.9672
			protocol:trial:compartment	11	428	0.67	0.7668
Figure 4B (KCs) and Suppl. Figure 3B	training-induced difference in response strength ~ trial*compartment*protocol, random = ~1|fly	all	(Intercept)	1	986	87.293	<.0001
			protocol	1	39	1.469	0.2328
			trial	1	986	30.548	<.0001
			compartment	10	275	17.36	<.0001
			protocol:trial	1	986	5.932	0.015
			protocol:compartment	10	275	5.705	<.0001
			trial:compartment	10	986	1.416	0.1678
			protocol:trial:compartment	10	986	0.802	0.6268
	training-induced difference in response strength ~ trial*protocol, random = ~1|fly	γ1	(Intercept)	1	64	1.424	0.2371
			protocol	1	20	3.713	0.0683
			trial	1	64	5.685	0.0201
			protocol:trial	1	64	1.682	0.1993
		γ2	(Intercept)	1	118	116.922	<.0001
			protocol	1	38	0.006	0.9364
			trial	1	118	8.429	0.0044
			protocol:trial	1	118	0.366	0.5465
		γ3	(Intercept)	1	121	151.995	<.0001
			protocol	1	39	1.296	0.262
			trial	1	121	2.102	0.1497
			protocol:trial	1	121	0.145	0.7045
		γ4	(Intercept)	1	121	41.275	<.0001
			protocol	1	39	0.248	0.6215
			trial	1	121	6.36	0.013
			protocol:trial	1	121	0.119	0.7306
		γ5	(Intercept)	1	121	55.269	<.0001
			protocol	1	39	1.107	0.2992

			trial	1	121	18.424	<.0001
			protocol:trial	1	121	0.002	0.9686
		β'1	(Intercept)	1	76	28.149	<.0001
			protocol	1	24	6.114	0.0209
			trial	1	76	0.764	0.3847
			protocol:trial	1	76	2.45	0.1217
		β'2	(Intercept)	1	121	35.53	<.0001
			protocol	1	39	11.52	0.0016
			trial	1	121	1.686	0.1966
			protocol:trial	1	121	4.357	0.039
		junction	(Intercept)	1	73	7.232	0.0089
			protocol	1	23	7.331	0.0126
			trial	1	73	3.076	0.0836
			protocol:trial	1	73	4.958	0.0291
		α1/α'1	(Intercept)	1	43	65.492	<.0001
			protocol	1	13	2.262	0.1565
			trial	1	43	0.997	0.3235
			protocol:trial	1	43	2.224	0.1432
		IPCs	(Intercept)	1	52	0.197	0.6592
			protocol	1	16	1.923	0.1845
			trial	1	52	2.356	0.1309
			protocol:trial	1	52	0.282	0.5977
		β2	(Intercept)	1	76	9.119	0.0034
			protocol	1	24	2.28	0.1441
			trial	1	76	0.444	0.5073
			protocol:trial	1	76	1.587	0.2116
Suppl. Figure 3D	training-induced difference in response strength ~ trial*compartment*protocol, random = ~1|fly	all	(Intercept)	1	314	13.732	0.0002
			protocol	1	39	0.847	0.3631
			trial	1	314	0.288	0.592
			compartment	10	275	3.912	0.0001
			protocol:trial	1	314	0.151	0.6979
			protocol:compartment	10	275	0.442	0.9251
			trial:compartment	10	314	1.869	0.0489
			protocol:trial:compartment	10	314	0.413	0.9401
Suppl. Figure 4C	electric current strength ~ pulse no.*trial*group, random = ~1|fly	NA	(Intercept)	1	1716	2254.346	<.0001
			pulse no.	1	1716	17.8124	<.0001
			group	3	60	0.8693	0.4621
			trial	1	1716	0.4487	0.503
			pulse no.:group	3	1716	4.6947	0.0029
			pulse no.:trial	1	1716	1.8692	0.1717
			group:trial	3	1716	1.2141	0.3031
			pulse no.:group:trial	3	1716	1.7119	0.1626

Suppl. Figure 5B	response strength ~ protocol*trial* concentration* compartment, random = ~1|fly	γ and β'	(Intercept)	1	436	2899328.4	<.0001
			protocol	1	10	0.7	0.4207
			trial	1	436	0.1	0.793
			concentration	1	436	10.6	0.0012
			compartment	7	436	164.5	<.0001
			protocol:trial	1	436	0.1	0.7025
			protocol:concentration	1	436	1.3	0.249
			trial:concentration	1	436	2.3	0.1307
			protocol:compartment	7	436	15.7	<.0001
			trial:compartment	7	436	0.3	0.9456
			concentration:compartment	7	436	0.2	0.9939
			protocol:trial:concentration	1	436	0	0.8956
			protocol:trial:compartment	7	436	0.2	0.9774
			protocol:concentration:compartment	7	436	0.1	0.9982
			trial:concentration:compartment	7	436	0	0.9999
			protocol:trial:concentration:compart-ment	7	436	0.1	0.9998
Figure 5B (CS vs. 1st CS)	angle between 9D-vectors ~ trial*protocol, random = ~1|fly	9 shared	(Intercept)	1	139	4.863	0.0291
			protocol	1	45	8.249	0.0062
			trial	1	139	9.909	0.002
			protocol:trial	1	139	1.062	0.3046
Figure 5B (US vs. 1st US)		9 shared	(Intercept)	1	92	597.721	<.0001
			protocol	1	45	0.109	0.7429
			trial	1	92	4.454	0.0375
			protocol:trial	1	92	0.105	0.747
Figure 5B (CS vs. mean US)		9 shared	(Intercept)	1	231	20.234	<.0001
			protocol	1	45	24.887	<.0001
			trial	1	231	11.234	0.0009
			protocol:trial	1	231	5.297	0.0223
Suppl. Figure 6A (CS vs. 1st CS)		9 shared	(Intercept)	1	121	222.493	<.0001
			protocol	1	39	1.528	0.2238
			trial	1	121	13.923	0.0003
			protocol:trial	1	121	3.569	0.0613
Suppl. Figure 6A (US vs. 1st US)		9 shared	(Intercept)	1	80	0.348	0.557
			protocol	1	39	0.881	0.3538
			trial	1	80	2.433	0.1227
			protocol:trial	1	80	0.444	0.5071
Suppl. Figure 6A (CS vs. mean US)		9 shared	(Intercept)	1	201	12.777	0.0004
			protocol	1	39	0.133	0.7172

			trial	1	201	5.661	0.0183
			protocol:trial	1	201	0.197	0.6573
Suppl. Figure 6B (CS vs. 1st CS)	Euclidean distance between 9D-vectors ~ trial*protocol, random = ~1|fly	9 shared	(Intercept)	1	139	271.208	<.0001
			protocol	1	45	0.547	0.4632
			trial	1	139	11.035	0.0011
			protocol:trial	1	139	2.011	0.1583
Suppl. Figure 6B (US vs. 1st US)		9 shared	(Intercept)	1	92	114.353	<.0001
			protocol	1	45	0.708	0.4044
			trial	1	92	0.963	0.3288
			protocol:trial	1	92	0.474	0.4925
Suppl. Figure 6B (CS vs. mean US)		9 shared	(Intercept)	1	233	88.191	<.0001
			protocol	1	45	11.317	0.0015
			trial	1	233	9.828	0.0019
			protocol:trial	1	233	7.336	0.0072
Suppl. Figure 6C (CS vs. 1st CS)		9 shared	(Intercept)	1	121	321.995	<.0001
			protocol	1	39	0.098	0.7548
			trial	1	121	13.216	0.0004
			protocol:trial	1	121	0.503	0.4792
Suppl. Figure 6C (US vs. 1st US)		9 shared	(Intercept)	1	80	1433.396	<.0001
			protocol	1	39	0.333	0.5667
			trial	1	80	5.195	0.0253
			protocol:trial	1	80	0.275	0.6010
Suppl. Figure 6C (CS vs. mean US)		9 shared	(Intercept)	1	203	161.300	<.0001
			protocol	1	39	1.134	0.2933
			trial	1	203	28.307	2.7249
			protocol:trial	1	203	3.095	0.0800
