# Supplementary material for: Trace Conditioning in Drosophila Induces Associative Plasticity in Mushroom Body Kenyon Cells and Dopaminergic Neurons
Source: Front Neural Circuits. 2017 Jun 20;11:42. doi: 10.3389/fncir.2017.00042 (PMC5476701; doi:10.3389/fncir.2017.00042)
Supplement: Supplementary file 2 [file Table2.pdf]

Supplementary Material

Trace conditioning in *Drosophila* induces associative plasticity in mushroom body Kenyon cells and dopaminergic neurons

Kristina V. Dylla[1], Georg Raiser[1], C. Giovanni Galizia[1], Paul Szyszka[1*]

[1] University of Konstanz, Department of Biology, Neurobiology, 78457 Konstanz, Germany

***Correspondence:**

Paul Szyszka

paul.szyszka@uni-konstanz.de

Supplementary Table 2. Related to Figure 3: DANs are more sensitive to current strength than KCs. Relationship between current flow through individual flies and induced responses in DANs and KCs. Spearman rank correlation was calculated between the current flow through each fly and the US-induced normalized neuronal responses. Additionally, the slope of the regression lines was calculated. Results and the number of flies (n) are listed for all regions for DANs and KCs. Non-availability of data is indicated by gray.

**MB compartments and other regions**	**DANs**				**KCs**			
	**p**	**ρ**	**slope of regression**	**n**	**p**	**ρ**	**slope of regression**	**n**
γ1	0	0.636	0.715	35	0.4746	0.175	0.027	19
γ2	0.0376	0.33	0.368	40	0.0661	0.305	0.021	37
γ3	0.0011	0.508	0.108	38	0.006	0.437	0.046	38
γ4	4.00E-04	0.547	0.196	38	0.4079	0.138	0.004	38
γ5	0.0017	0.481	0.207	40	0.2379	0.196	0.013	38
β'1	0.0091	0.454	0.178	32	0.0401	0.422	0.047	24
β'2	8.00E-04	0.51	0.161	40	0.0212	0.373	0.022	38
junction	0.0052	0.475	0.321	33	0.5939	0.12	0.013	22
α1/α'1	0.879	-0.071	0.024	7	0.0666	0.503	0.052	14
FB1	0.0056	0.486	0.215	31				
FB2	0.0107	0.475	0.282	28				
EB	0.0044	0.583	0.414	22				
β2					0.0567	0.485	0.519	16
IPCs					0.4115	0.18	0.012	23
