# Supplementary material for: Trace Conditioning in Drosophila Induces Associative Plasticity in Mushroom Body Kenyon Cells and Dopaminergic Neurons
Source: Front Neural Circuits. 2017 Jun 20;11:42. doi: 10.3389/fncir.2017.00042 (PMC5476701; doi:10.3389/fncir.2017.00042)
Supplement: Supplementary file 3 [file DataSheet1.pdf]

Supplementary Material

Trace conditioning in *Drosophila* induces associative plasticity in mushroom body Kenyon cells and dopaminergic neurons

Kristina V. Dylla[1], Georg Raiser[1], C. Giovanni Galizia[1], Paul Szyszka[1*]

[1] University of Konstanz, Department of Biology, Neurobiology, 78457 Konstanz, Germany

***Correspondence:**

Paul Szyszka

paul.szyszka@uni-konstanz.de

Supplementary Experimental Procedures

Flies and fly preparation

Flies: We reared flies on standard cornmeal medium at 25°C and 60 % rel. humidity under a 12:12 hours light:dark cycle. For visualizing neuronal activity, we used the GAL4-UAS system for targeted gene expression (Brand and Perrimon 1993) to express the calcium-sensitive fluorescent protein GCaMP3 in DANs or KCs. All experimental flies were 1 – 11 days old F1 females (average age: 5 days).

Fly preparation: We fixed the flies with wax in a plastic holder such that the fly's dorsal side could be bathed in saline while its ventral side in air (including antennae and legs) was accessible for odorant and electric shock application (Figure 2A). In *TH>GCaMP3* flies, we fixed the proboscis with wax to reduce brain movement. After sealing the preparation we cut open the fly head dorsally and covered the preparation with saline (130 mM NaCl, 5 mM KCl, 2 mM $MgCl_2$, 2 mM $CaCl_2$, 36 mM sucrose, and 5 mM HEPES; pH 7.3). As a last step we removed glands and trachea from the brain.

Stimuli and stimulus control

Electric shock: We applied electric shocks to the fly's legs by placing the fly on a custom-build copper grid, such that right- and left-side legs were touching opposing electric poles (Figure 2A, Supplementary Figure 4B). Since the fly could move its legs, the received electric shock strength varied. Therefore, we recorded the shock strength received by an individual fly using a bridge circuit (sampling rate: 16 kHz; Figure 3A and Supplementary Figure 4C). We measured the mean voltage change (U_{def}) during each shock pulse by subtracting the pre-stimulus voltage (mean of 200 – 100 ms before shock) and excluded cases where this value was negative. We calculated the current through the fly as $I_{fly} = \frac{U_{def} *(R_{def} + R_{osc})}{R_{def} * R_{osc}}$. We tested for significance in electric shock strength differences between the four experimental groups (Supplementary Figure 4C) using a linear mixed-effect model on log-transformed values (R: "lme" function; for the results see Table S1).

Odorant stimuli: We used 1-butanol (BUT; Fluka, CAS 71-36-3) and 4-methylcyclohexanol (MCH; Sigma-Aldrich, CAS 589-91-3) diluted in mineral oil (MO; Sigma-Aldrich, CAS 8042-47-5; BUT 1:500, MCH 1:1000). We prepared 10 ml odorant solutions in 100 ml glass bottles. We stimulated with 10-second-long odorant stimuli using a custom-build olfactometer (Szyszka et al. 2011) with a continuous air flow (1.3 m/s, 43 % rel. humidity). For measuring odorant stimulus dynamics, we positioned the inlet of a photo ionization detector (miniPID, Modell 200B, Aurora Scientific Inc) at the location of the fly, and kept everything else identical to the imaging experiments. We baseline-corrected the data and down-sampled it to 10 Hz by taking the mean over 50 data points (Supplementary Figure 4A).

We adopted the stimulation protocols ("paired" and "unpaired", Figure 1) from Galili and colleagues (Galili, et al. 2011). During pre-training we presented the solvent (MO), the olfactory CS (BUT, without US) and the control odorant (MCH). We trained with six CS - US pairing trials in which the olfactory CS was followed by the electric shock US (interval between the onsets of CS and US: 15 s, stimulus-free gap: 5 s). The unpaired training consisted of six trials of CS and US, in which CS and US were separated by 80 s, and in which the sequence of CS and US was pseudorandomized. We kept the imaging times equal for the paired and unpaired protocol by recording only the first stimulus in each of the unpaired training trials. During post-training we measured the responses to the CS, the control odorant and the US. To confirm that the fly was still alive, we presented a very last CS at the end of the stimulation protocol. Each stimulation protocol lasted 45.5 minutes.

Calcium imaging

We measured the fluorescence of GCaMP3 and DsRed with a confocal laser scanning microscope (LSM 510 META, Zeiss), equipped with a 20x water-immersion objective (W-PlanApochromat 20x DIC VIS-IR, numerical aperture 1.0, Zeiss). We used a 488 nm argon laser line with a 500 – 530 nm emission filter for GCaMP3, and a 535 – 590 nm emission filter for DsRed. We acquired images at 12 bit depth and 5 Hz with 68 x 131 pixels resolution, corresponding to an area of 204 x 393 µm in the fly brain. Optical slices were 10 µm for recordings of KCs and 15 µm for recordings of DANs. When possible, we recorded simultaneously the γ2 compartment of the MB-lobes in both hemispheres (Figure 2B). Additionally, we sought for a good visibility of the separation of γ- and β'-lobe. When necessary, we readjusted the focus after each recording period. Each recording lasted 45 s, followed by a 165 s non-recording period (Figure 1).

Data analysis

Imaging data: We conducted an automatized movement correction (source code available at https://github.com/grg2rsr/xyt_movement_correction), which for DANs was based on the DsRed signal. We further conducted a manual movement correction between trials. For the calculation of $\Delta F/F_0$ we subtracted the background fluorescence before odorant onset (F_0, mean of frames 3 – 24) from each frame. For normalizing the $\Delta F/F_0$-traces, we divided the $\Delta F/F_0$-values by the maximum response to the CS in trial 2 within each fly, irrespective of which region showed the strongest response.

Identification of MB-compartments: We based our visual identification of the MB-compartments on anatomical studies by Tanaka et al. (2008) and Aso et al. (2014). For this purpose, we used the GCaMP3- and DsRed-expression for KCs and DANs, respectively. To minimize the risk of mixing up the signals of neighboring compartments we defined the borders for each compartment in a conservative manner and drew the shape for each compartment

smaller than we assumed the actual compartment to be. Outlines for different compartments never overlapped (see an example in Figure 2B, bottom).

Correlation of electric shock strength and neuronal responses (Figure 3B, C and Table S2): We tested the neuronal sensitivity to electric shock strength by correlating the mean electric current (for the calculation see above) with the normalized mean calcium responses of DANs and KCs during the very first shock pulse in trial 4, referred to as “mean response”. We analyzed each compartment individually using Spearman's correlation (R: “cor.test” function).

Color-coded images (Figure 2C and Figure 5A): We calculated the percentage of mean $\Delta F/F_0$ during stimulus application which was 10 s for odorants and 4 x 1.5 s for electric shocks. For KCs, we defined all raw fluorescence values below 300 as background and set them to 0.

Changes in response trace (Figure 4A): The color-coded p-values above the response traces were calculated with the “wilcox.test” function in R.

Spatial activity patterns (Figure 5B and Supplementary Figure 6): The dissimilarity between two spatial activity patterns was determined by the Euclidean distance or the angle (φ) between the two respective vectors ($\vec{\alpha}$ and $\vec{\beta}$).

$$\cos \varphi = \frac{\vec{\alpha} \circ \vec{\beta}}{||\vec{\alpha}||\ ||\vec{\beta}||}$$

First, we calculated the Euclidean distance or the angle between the respective vectors for each fly. Second we averaged these Euclidean distances or angles across flies. Different than the Euclidean distance, the angle is not influenced by uniform changes in activity strength but it is affected by the ratio of activity in the compartments. Angles can vary between 0 rad (0°; same vector direction) and π rad (180°; opposite vector direction). To test for training-related changes in the pattern induced by the CS, we compared each CS-induced pattern against pre-training. Accordingly, we compared the US-induced patterns against the naïve pattern in trial 4. We also probed the similarity between US- and CS-induced patterns by comparing the mean response to the US over the trials 4, 7, 9 and 12 (available for both treatment groups) against the patterns induced by the CS in individual trials. We tested for differences in pattern dissimilarity between paired and unpaired group using a linear mixed-effect model on log-transformed values (R: “lme” function).

Software: We created a mask of all regions of interest in Photoshop CS4 (Adobe). We retrieved the fluorescence values for all regions of interest using IDL. Further data processing and analysis we conducted in R (version i386 3.1.2, (R Core Team 2014)) using custom-written routines and the following packages: “reshape” (Wickham 2007), “scales” (Wickham 2012), “gridExtra” (Auguie 2012), “ggplot2” (Wickham 2009), “tidyr” (Wickham 2014), “dplyr” (Wickham and Francois 2016), “forecast” (Hyndman and Khandakar 2007), and “nlme” (Pinheiro et al., 2013). For schematic drawings we used Illustrator CS2 (Adobe).

Statistics: To meet the criteria for parametric statistical methods we had to use a Box-Cox transformation (R: “BoxCox” function of the “forecast” package (Hyndman and Khandakar 2007) on the DAN data to achieve normal distribution. We tested for differences between hemispheres, between experimental groups, between concentrations, between shock pulses, trials, and between paired and unpaired group using linear mixed-effect models (R: “lme” function of the “nlme” package (Pinheiro et al., 2013). To test for a correlation between neuronal response strength and received electric current, we used Spearman’s test (R: “cor.test” function).

Supplementary References

Aso, Y., Hattori, D., Yu, Y., Johnston, R. M., Iyer, N. A., Ngo,T. T. B., et al. (2014) The Neuronal Architecture of the Mushroom Body Provides a Logic for Associative Learning. *eLife* 3 (December): e04577. doi:10.7554/eLife.04577.

Auguie, B. (2012) gridExtra: Functions in Grid Graphics (R Package). http://cran.r-project.org/web/packages/gridExtra/index.html.

Brand, A. H., and Perrimon, N. (1993) Targeted Gene Expression as a Means of Altering Cell Fates and Generating Dominant Phenotypes. *Development (Cambridge, England)* 118 (2): 401–15. http://www.ncbi.nlm.nih.gov/pubmed/8223268.

Galili, D. S., Lüdke, A., Galizia, C. G., Szyszka, P., and Tanimoto, H. (2011) Olfactory Trace Conditioning in *Drosophila*. *The Journal of Neuroscience : The Official Journal of the Society for Neuroscience* 31 (20): 7240–48. doi:10.1523/JNEUROSCI.6667-10.2011.

Hyndman, R. J., and Khandakar, Y. (2007) Automatic Time Series Forecasting: The Forecast Package for R. http://www.buseco.monash.edu.au/depts/ebs/pubs/wpapers/.

Pinheiro, J., Bates, D., DebRoy, S., Sarkar, D., R Core Team (2013) Nlme: Linear and Nonlinear Mixed Effects Models. *Http://cran.r-Project.org/package=nlme*.

R Core Team (2014) A Language and Environment for Statistical Computing. http://www.r-project.org/.

Raiser, G., Galizia, C. G., and Szyszka, P. (2017) A High-Bandwidth Dual-Channel Olfactory Stimulator for Studying Temporal Sensitivity of Olfactory Processing. *Chemical Senses* 42 (2): 141–51. doi:10.1093/chemse/bjw114.

Szyszka, P. Demmler, C., Oemisch, M., Sommer, L., Biergans, S., Birnbach, B., Silbering, A. F., and Galizia, C. G. (2011) Mind the Gap: Olfactory Trace Conditioning in Honeybees. *The Journal of Neuroscience* 31 (20): 7229–39. doi:10.1523/JNEUROSCI.6668-10.2011.

Tanaka, N. K., Tanimoto, H., and Ito, K. (2008) Neuronal Assemblies of the *Drosophila* Mushroom Body. *The Journal of Comparative Neurology* 508 (5): 711–55. doi:10.1002/cne.21692.

Wickham, H. (2014) Tidyr: Easily Tidy Data with Spread and Gather Functions. *R Package Version 0.1, URL http://CRAN. R-Project. Org/package= Tidyr*. https://github.com/hadley/tidyr/.

Wickham, H. (2012) Scales: Scale Functions for Graphics. *http://CRAN.R-Project.org/package=scales*.

Wickham, H. (2007) Reshaping Data with the Reshape Package. *JSS Journal of Statistical Software* 21 (12). http://www.jstatsoft.org/.

Wickham, H. (2009) *Ggplot2 : Elegant Graphics for Data Analysis*. Springer.

Wickham, H., and Francois, R. (2016) Dplyr: A Grammar of Data Manipulation. R Package Version 0.5.0. https://rdrr.io/cran/dplyr/man/dplyr.html.
