# Supplementary material for: Trace Conditioning in Drosophila Induces Associative Plasticity in Mushroom Body Kenyon Cells and Dopaminergic Neurons
Source: Front Neural Circuits. 2017 Jun 20;11:42. doi: 10.3389/fncir.2017.00042 (PMC5476701; doi:10.3389/fncir.2017.00042)
Supplement: Supplementary file 4 [file DataSheet2.pdf]

Supplementary Material

Trace conditioning in *Drosophila* induces associative plasticity in mushroom body Kenyon cells and dopaminergic neurons

Kristina V. Dylla[1], Georg Raiser[1], C. Giovanni Galizia[1], Paul Szyszka[1*]

[1] University of Konstanz, Department of Biology, Neurobiology, 78457 Konstanz, Germany

***Correspondence:**

Paul Szyszka

paul.szyszka@uni-konstanz.de

Supplementary Figures

Supplementary Figure 1. Related to Figure 2 and Figure 4: DAN response traces obtained for paired CS-US presentation for all regions. Normalized DAN response traces obtained for the paired protocol for the regions γ1 – 5, β'1 – 2, junction, α1/α1', fan-shaped body (FB) 1 – 2, and ellipsoid body (EB). Traces represent the median and quartiles (number of flies (n) is indicated in the figure).

Supplementary Figure 2. Related to Figure 2 and Figure 4: KC response traces obtained for paired CS-US presentation for all regions. Normalized KC response traces obtained for the paired protocol for the regions γ1 – 5, β'1 – 2, β2, junction, α1/α1', and insulin-producing cells (IPCs). Traces represent the median and quartiles (number of flies (n) is indicated in the figure).

Supplementary Figure 3. Related to Figure 4: Associative plasticity in DAN and KC responses. (A, B) CS-induced response strength in DANs (A) and KCs (B) during the six training trials (green background) and the post-training for the paired (red) and the unpaired (black) group. The pre-training response strength to either the CS or the control odorant was subtracted from each value. **(A)** In DANs, during training the CS-induced response strength increased in the paired group relative to the unpaired group for the regions γ1 – 5, β'1 – 2, and the junction (mixed-effect model for repeated-measures ANOVA). Post-training the CS-induced response strength was higher in the paired than in the unpaired group in those regions, but for the response strength induced by the control odorant (blue background) there was no significant difference between the paired and unpaired group. **(B)** In KCs, during training the CS-induced response strength decreased in the paired group relative to the unpaired group for the regions β'1 – 2, and the junction (mixed-effect model for repeated-measures ANOVA). Post-training the CS-induced response strength was lower in the paired than in the unpaired group in those regions, but for the response strength induced by the control odorant (blue background) there was no significant difference between the paired and unpaired group. **(C, D)** US-induced response strength in DANs (C) and KCs (D) during the six training trials and the post-training for the paired (red) and the unpaired (black) group. The US-induced response strength obtained for the first training trial was subtracted from each value. **(C)** In DANs, during

training the US-induced response strength decreased similarly in the paired and unpaired group in all regions of interest (mixed-effect model for repeated-measures ANOVA: p = 0.082). **(D)** In KCs, during training the US-induced response strength decreased similarly in the paired and unpaired group in all regions of interest (mixed-effect model for repeated-measures ANOVA: p = 0.363). Number of flies (n) and p-values are indicated in the figure. **(E)** Relationship between the strength of the received electric shock in the first training trial and the change of CS-induced DAN responses in the second training trial. Response change did not correlate with the received current (red regression line). Results of a Spearman rank correlation test (ρ, p) and the number of flies (n) are indicated above the scatter plots.

Supplementary Figure 4 Related to Figure 3 and Figure 4: Stimulus monitoring. (A) The CS terminates before US onset. Normalized photo ionization detector (PID) signals induced by the CS (top) and the control odorant (bottom) for pre-training (2, 3), training (4 – 9), and post-training (10, 11, 13) trials. The maximum PID signal during pre-training was set to 100 %. After CS offset the CS concentration returns to zero (horizontal red line) before the US starts (vertical red line). All traces represent the median and quartiles (n = 5). **(B)** Photo of the fixed fly in the recording set up. **(C)** Electric current received by flies during US presentations was similar among groups. Electric current flow during single US pulses in each experimental group. Top: electric current flow during DAN imaging in the paired (red) and unpaired (black) group. Bottom: electric current flow during KC imaging in the paired and unpaired group. The US was applied as a train of four pulses in each of the training trials (4 – 9) and in the post-training trial (12). All data is displayed as boxplots (middle line in box represents the median, lower box bound the first quartile, upper box bound the third quartile, whiskers the 95 % confidence interval of the mean, and dots represent outliers of the 95 % confidence interval; mixed-effect model for repeated-measures ANOVA: p = 0.462). Number of flies (n) is indicated in the figure.

Supplementary Figure 5. Related to Figure 4: DANs are sensitive to odorant concentration and stimulus sequence. (A) PID signals for 1-butanol stimuli. Bars below the signals indicate the 10-second-long opening of the valve for each stimulus. Left: initial 1-butanol stimulus which we used as CS in the paired and unpaired groups, diluted 1:500 in mineral oil (BUT, n = 1). Middle: 1-butanol stimulus produced by mixing the headspace of pure 1-butanol with air, with mixing ratios adjusted to match the concentration of the initial BUT (high concentration, H; mean (black) of 3 measurements (grey)). Repeated measurements of the same stimulus show a stable 1-butanol concentration. Right: Same as H, but the 1-butanol concentration was adjusted to 60 % BUT (low concentration, L), corresponding to the total drop in concentration over the course of an experiment. H and L were presented with the olfactory stimulator used in (Raiser, Galizia, and Szyszka 2017). The air speed at the stimulator outlet matched the airspeed at the outlet of the stimulator used in the rest of the paper. The interstimulus interval of 1-butanol stimuli was 45 seconds. **(B)** Calcium response strength of DANs during alternating presentations of 100 % BUT (H) and 60 % BUT (L). The first-trial response strength was subtracted in all trials. DANs' response strength was generally stronger for H than for L (mixed-effect model for repeated-measures ANOVA, p < 0.01), and in some compartments depended on the 1-butanol concentration the protocol started with (H or L) (p < 0.001). Calcium responses were recorded in F1 flies obtained from crossing females homozygous for *UAS-GCaMP6f* (Chen et al., 2013) with males homozygous for *mb247-DsRed* and *TH-GAL4*.

Supplementary Figure 6. Related to Figure 5: Odor - shock conditioning does not affect the KCs spatial activity patterns. (A) Dissimilarity of spatial activity patterns in KCs, quantified as the angle between two vectors that comprise the response strengths of nine MB compartments. Training similarly changed the CS-induced spatial activity pattern in the paired

(red) and unpaired (black) group. CS vs. 1st CS: during training the CS-induced spatial activity patterns became dissimilar to the pre-training spatial activity pattern. This effect was equally strong in the unpaired and in the paired group. US vs. 1st US: during training the US-induced spatial activity patterns became equally dissimilar to the first US-induced spatial activity pattern in the paired and unpaired group. CS vs. mean US: during training the degree of dissimilarity between CS- and US-induced spatial activity patterns remained unchanged for the paired and for the unpaired group. **(B)** Dissimilarity of spatial activity patterns in DANs, quantified as the Euclidean distance between two vectors that comprise the response strengths of nine MB compartments. **(C)** Dissimilarity of spatial activity patterns in KCs, quantified as the Euclidean distance. All values represent the mean and SEM (p-values and number of flies (n) are indicated in the figure; mixed-effect model for repeated-measures ANOVA (see Supplementary Table 1 for details).
