# Supplementary figures and images for: Trace Conditioning in Drosophila Induces Associative Plasticity in Mushroom Body Kenyon Cells and Dopaminergic Neurons
Source: Front Neural Circuits. 2017 Jun 20;11:42. doi: 10.3389/fncir.2017.00042 (PMC5476701; doi:10.3389/fncir.2017.00042)

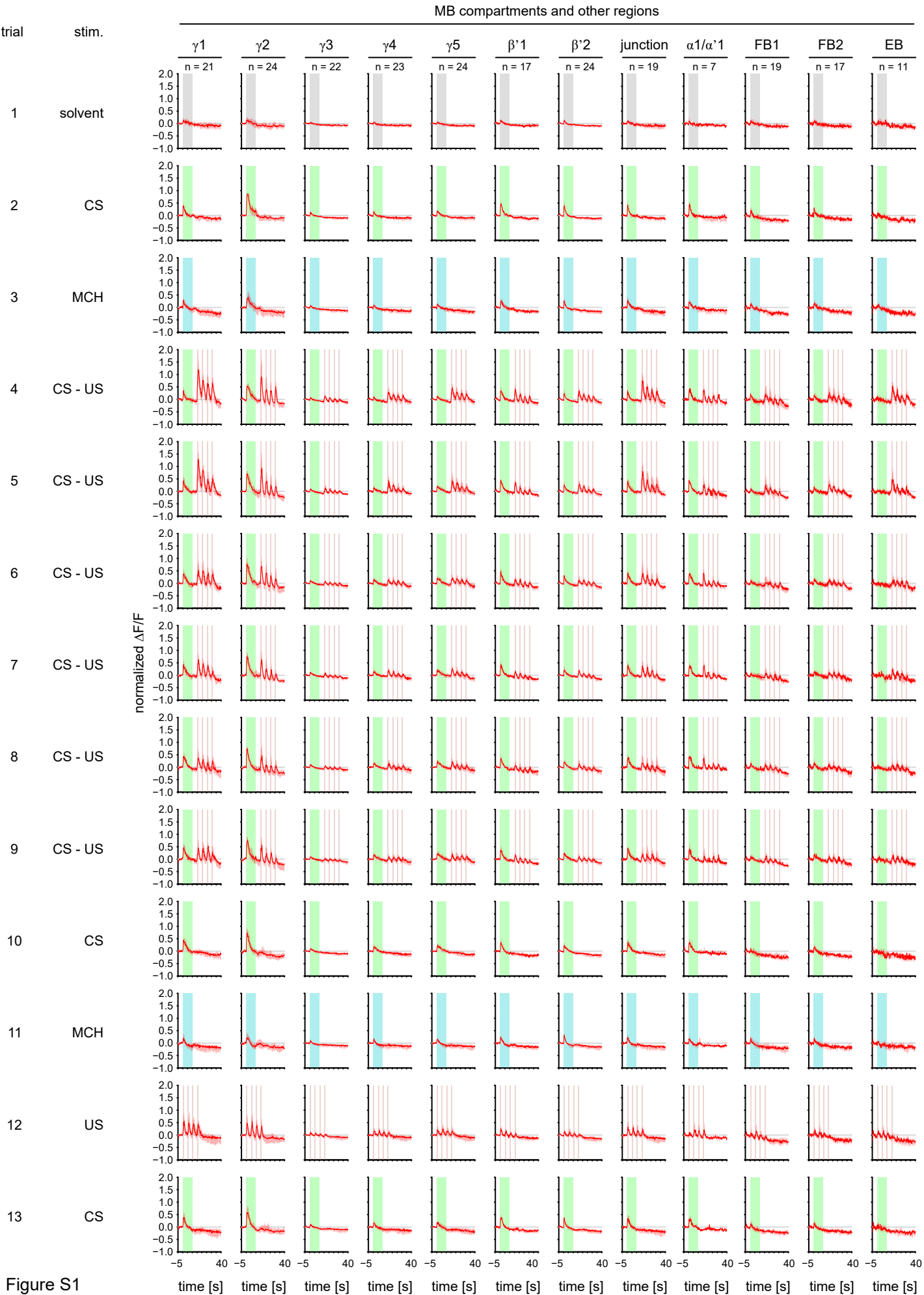

Supplement: Supplementary file 5 [file Image1.PDF]

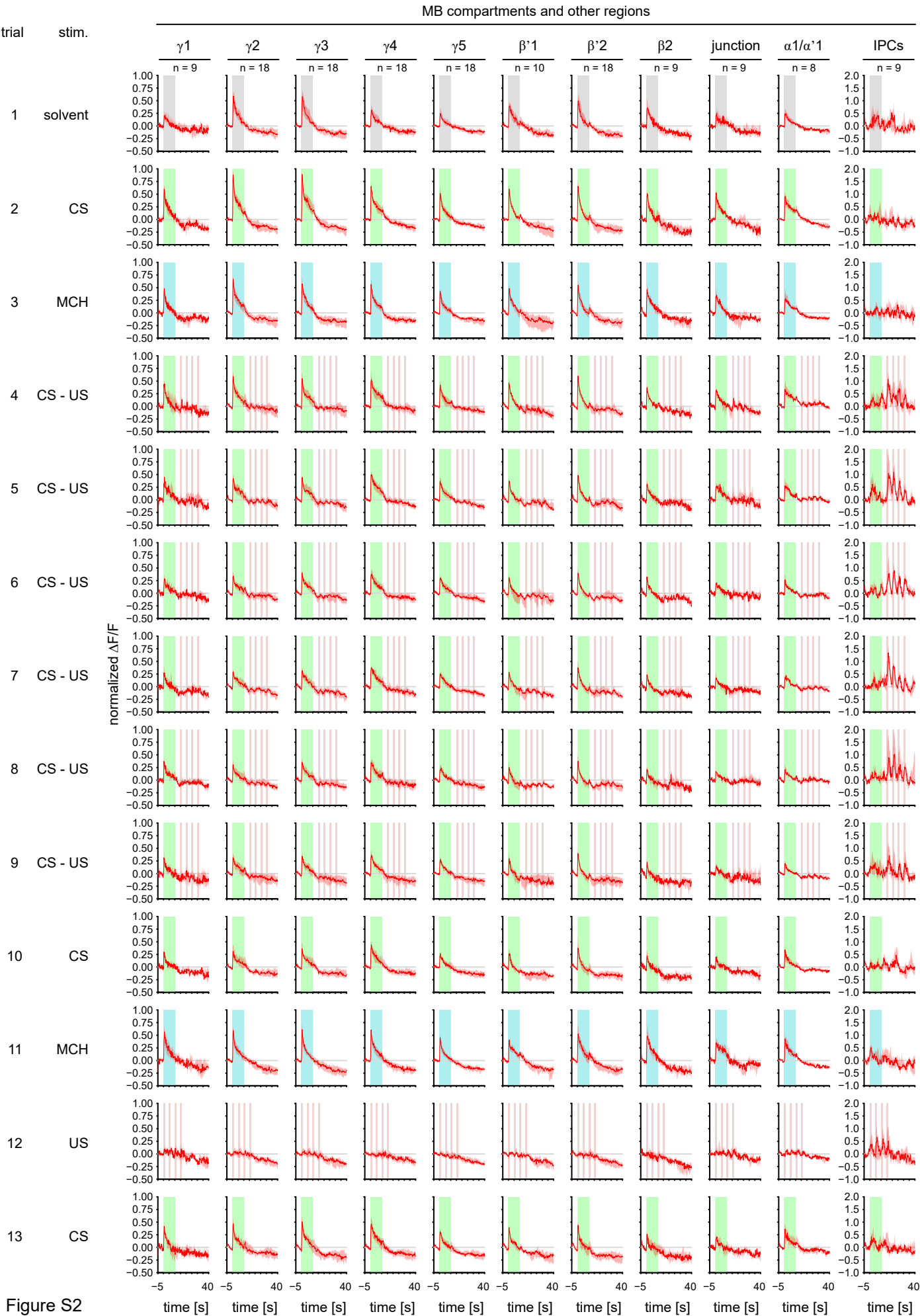

Supplement: Supplementary file 6 [file Image2.PDF]

A

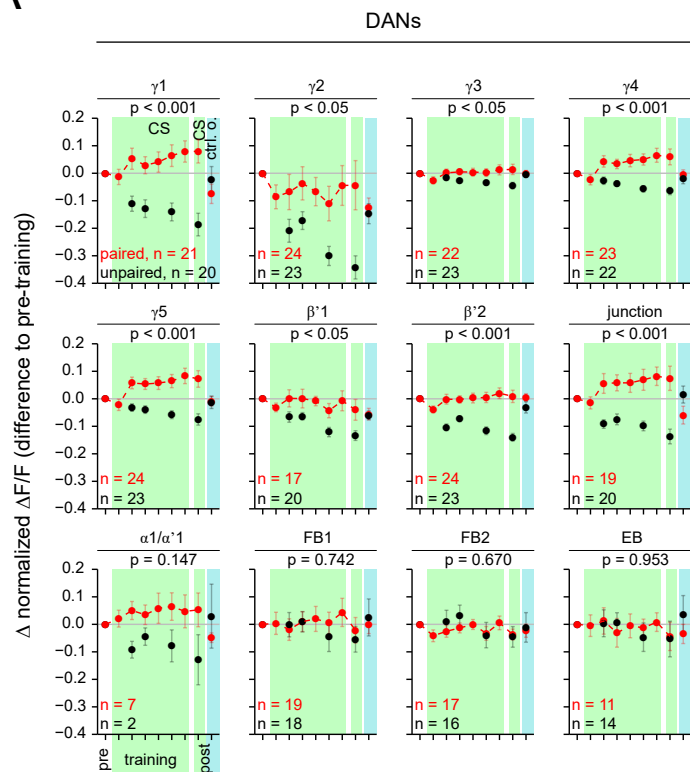

B

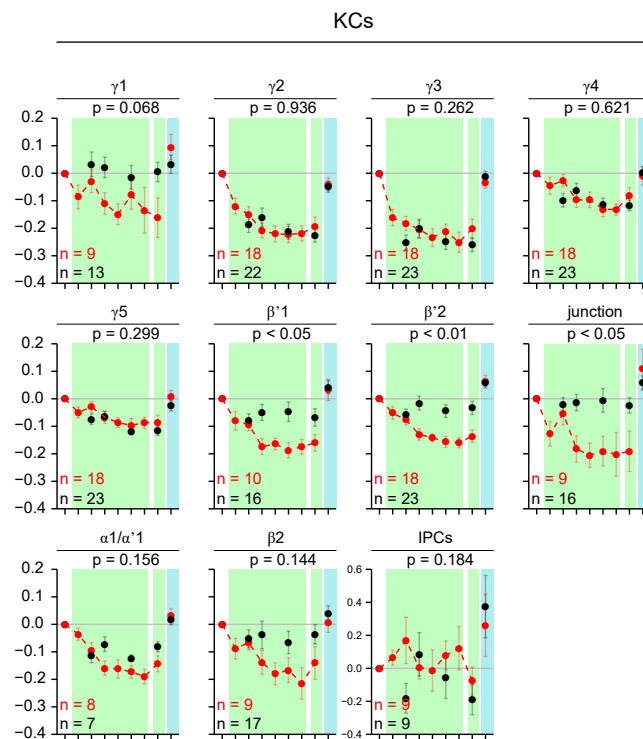

C

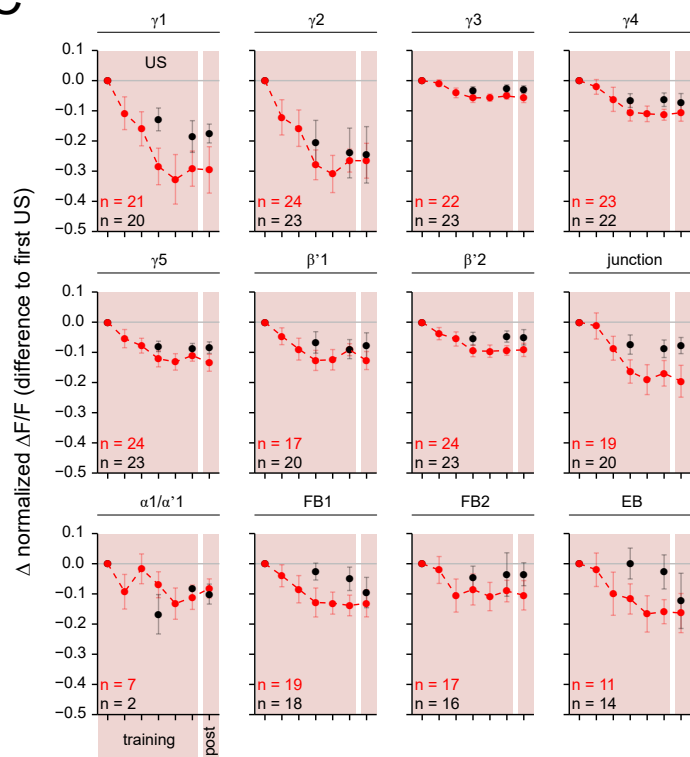

D

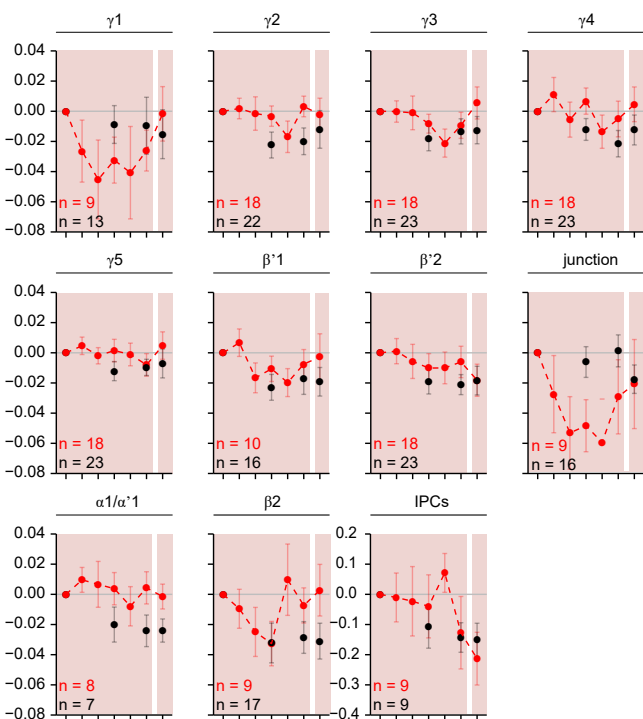

E

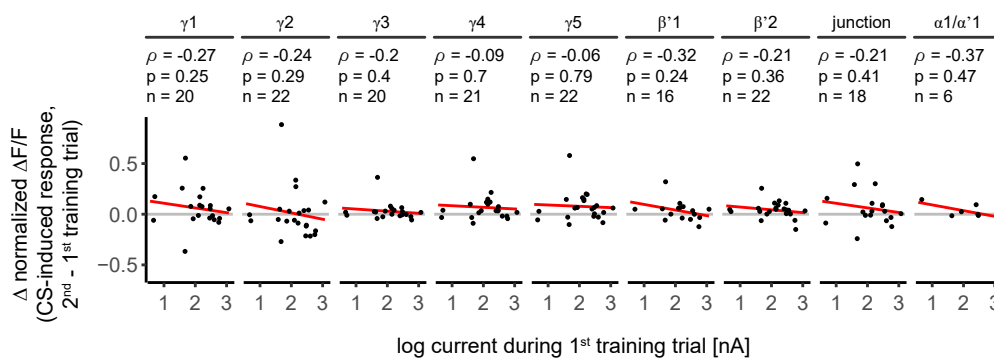

Figure S3

Supplement: Supplementary file 7 [file Image3.PDF]

A

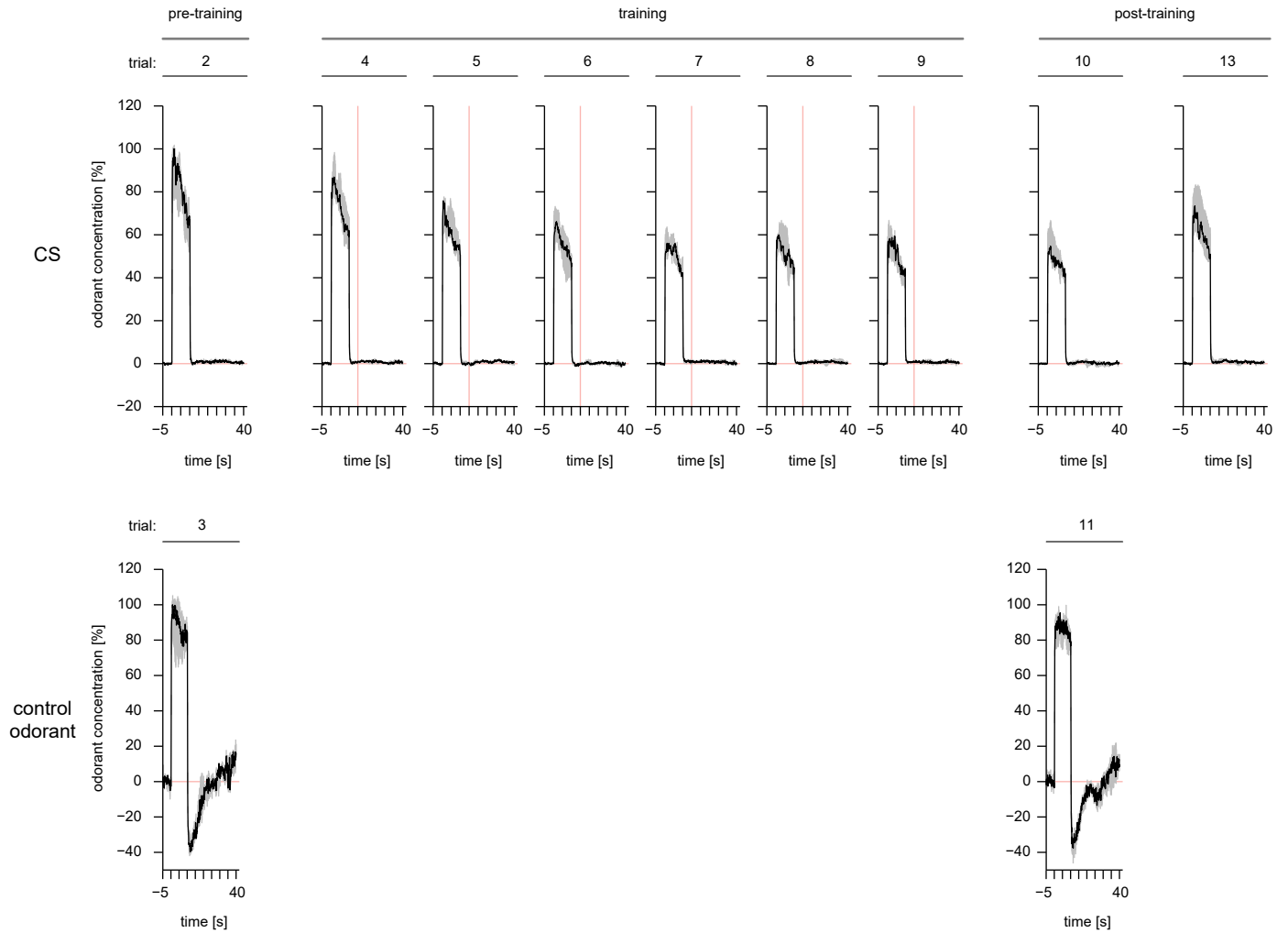

B

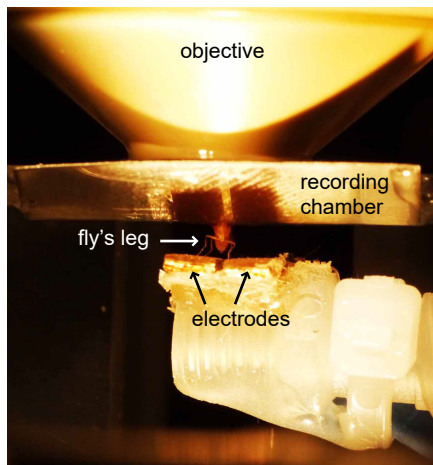

C

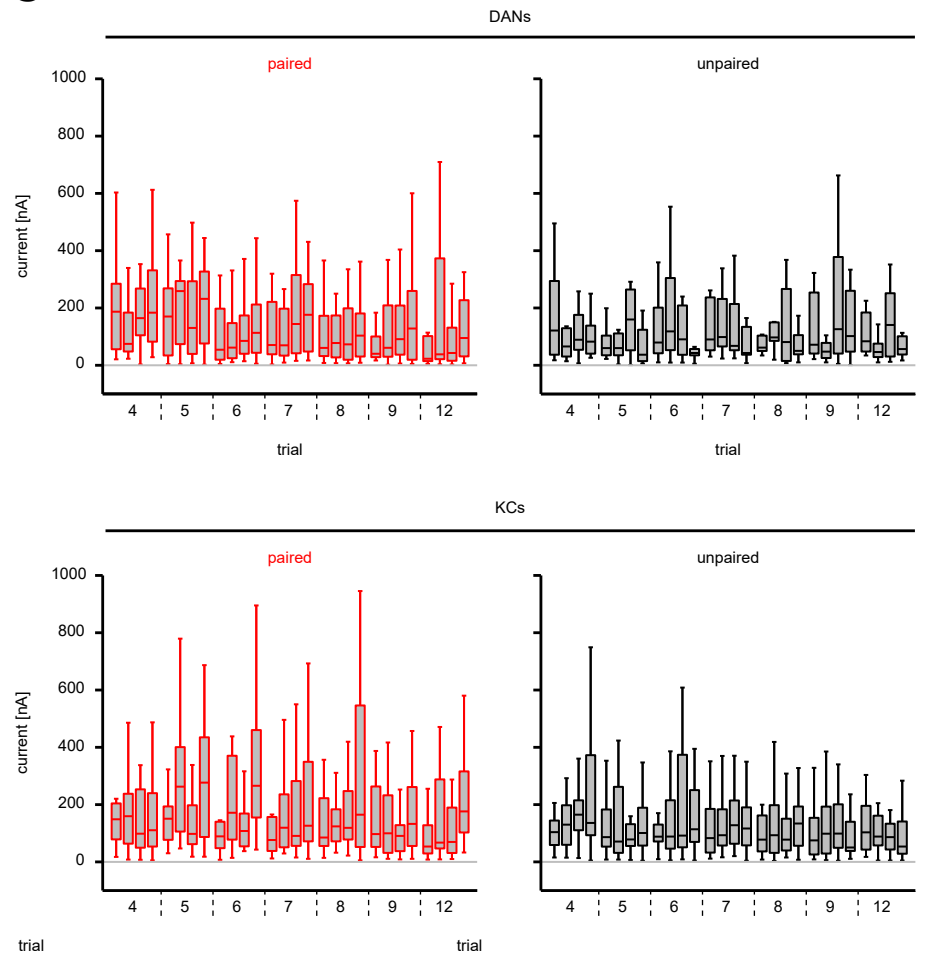

Figure S4

Supplement: Supplementary file 8 [file Image4.PDF]

A

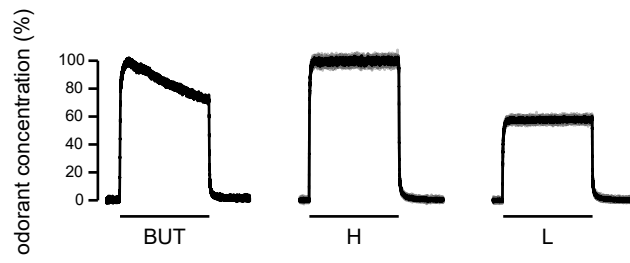

B

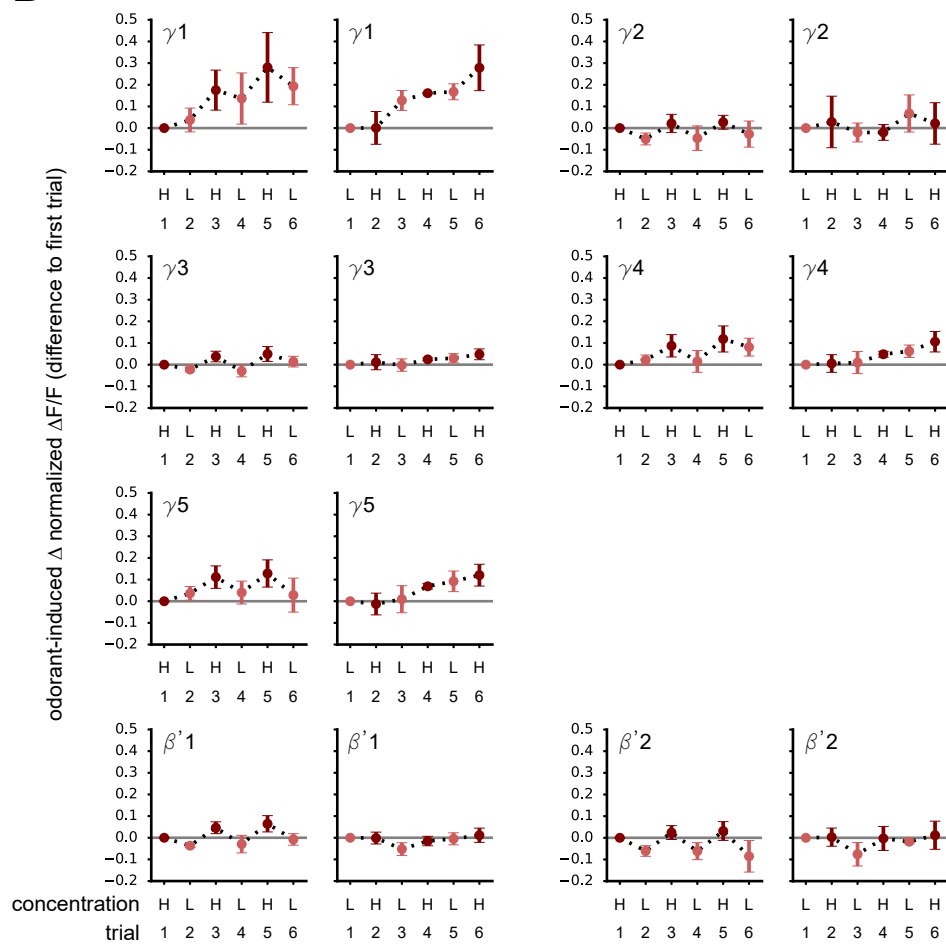

Figure S5

Supplement: Supplementary file 9 [file Image5.PDF]

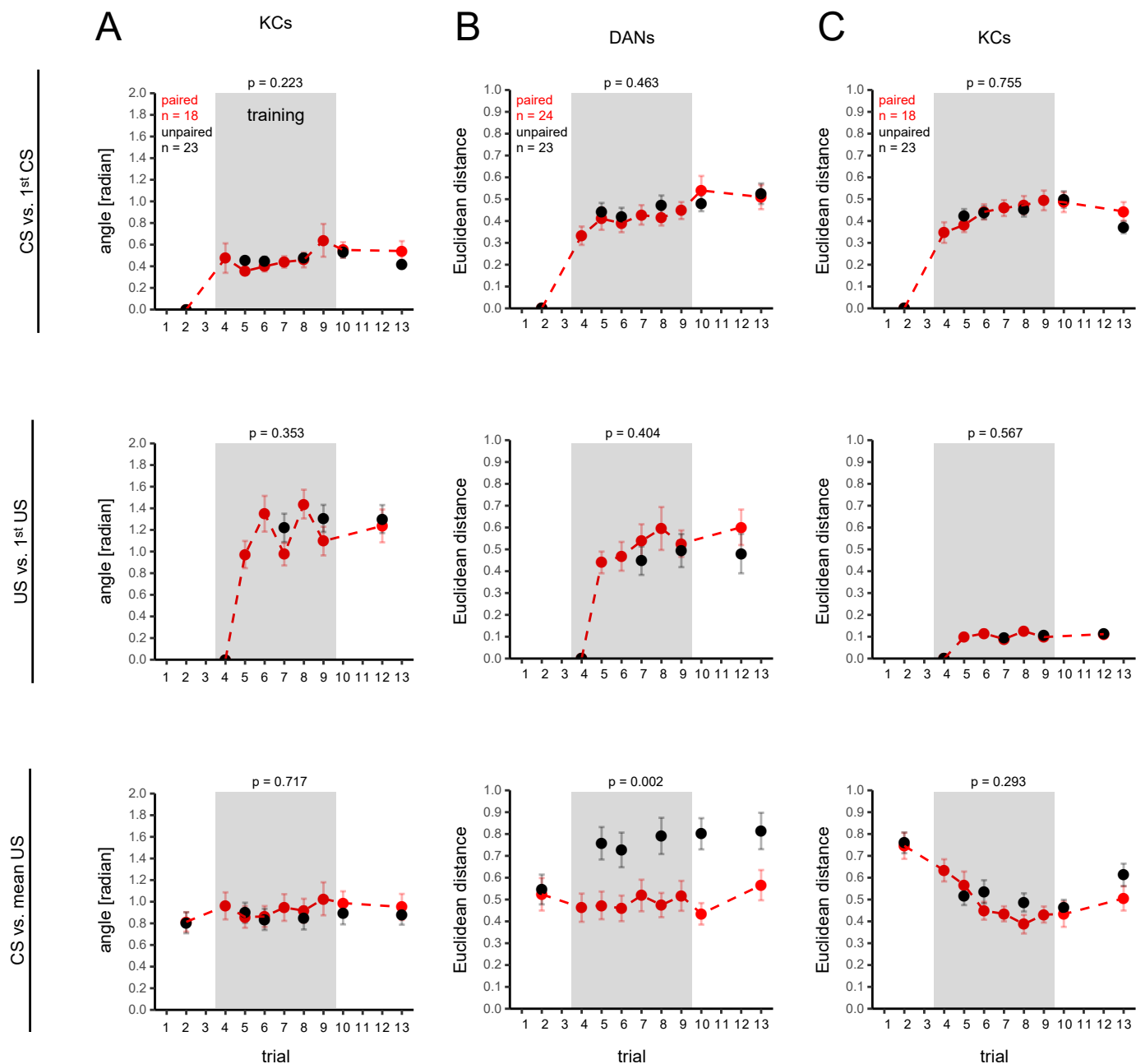

Figure S6

Supplement: Supplementary file 10 [file Image6.PDF]
